# A conserved KLF-autophagy pathway modulates nematode lifespan and mammalian age-associated vascular dysfunction

**DOI:** 10.1038/s41467-017-00899-5

**Published:** 2017-10-13

**Authors:** Paishiun N. Hsieh, Guangjin Zhou, Yiyuan Yuan, Rongli Zhang, Domenick A. Prosdocimo, Panjamaporn Sangwung, Anna H. Borton, Evgenii Boriushkin, Anne Hamik, Hisashi Fujioka, Ciaran E. Fealy, John P. Kirwan, Maureen Peters, Yuan Lu, Xudong Liao, Diana Ramírez-Bergeron, Zhaoyang Feng, Mukesh K. Jain

**Affiliations:** 1 Department of Medicine, Case Cardiovascular Research Institute, Case Western Reserve University, 10900 Euclid Avenue, Cleveland, OH 44106 USA; 2 Harrington Heart and Vascular Institute, University Hospitals Case Medical Center, 2103 Cornell Road, Cleveland, OH 44106 USA; 3 Department of Pathology, Case Western Reserve University, 10900 Euclid Avenue, Cleveland, OH 44106 USA; 4 Department of Pharmacology, Case Western Reserve University, 10900 Euclid Avenue, Cleveland, OH 44106 USA; 5 Department of Physiology and Biophysics, School of Medicine, Case Western Reserve University, 10900 Euclid Avenue, Cleveland, OH 44106 USA; 6 Electron Microscopy Facility, 10900 Euclid Avenue, Cleveland, OH 44106 USA; 7 Department of Pharmacology, Center for Mitochondrial Diseases, 10900 Euclid Avenue, Cleveland, OH 44106 USA; 8 Department of Biomedical Sciences, Kent State University, Cunningham Hall, Kent, OH 44242 USA; 9 Department of Pathobiology, Lerner Research Institute, 9500 Euclid Avenue, Cleveland Clinic, Cleveland, OH 44195 USA; 10 Metabolic Translational Research Center, Cleveland Clinic Foundation, 9500 Euclid Avenue/ M83-02, Cleveland, OH 44195 USA; 11 Department of Biology, Oberlin College, 119 Woodland Street, Oberlin, OH 44074 USA

## Abstract

Loss of protein and organelle quality control secondary to reduced autophagy is a hallmark of aging. However, the physiologic and molecular regulation of autophagy in long-lived organisms remains incompletely understood. Here we show that the Kruppel-like family of transcription factors are important regulators of autophagy and healthspan in *C. elegans*, and also modulate mammalian vascular age-associated phenotypes. Kruppel-like family of transcription factor deficiency attenuates autophagy and lifespan extension across mechanistically distinct longevity nematode models. Conversely, Kruppel-like family of transcription factor overexpression extends nematode lifespan in an autophagy-dependent manner. Furthermore, we show the mammalian vascular factor Kruppel-like family of transcription factor 4 has a conserved role in augmenting autophagy and improving vessel function in aged mice. Kruppel-like family of transcription factor 4 expression also decreases with age in human vascular endothelium. Thus, Kruppel-like family of transcription factors constitute a transcriptional regulatory point for the modulation of autophagy and longevity in *C. elegans* with conserved effects in the murine vasculature and potential implications for mammalian vascular aging.

## Abstract (web summary)

KLF family transcription factors (KLFs) regulate many cellular processes, including proliferation, survival and stress responses. Here, the authors position KLFs as important regulators of autophagy and lifespan in *C. elegans*, a role that may extend to the modulation of age-associated vascular phenotypes in mammals.

## Introduction

The maintenance of cellular and organismal homeostasis determines the progress of aging. On a cellular level, homeostasis is maintained, in part, through macroautophagy (hereafter referred to as autophagy), a conserved mechanism by which a cell achieves multiple goals, including clearance of misfolded proteins and organelle turnover with subsequent recycling of degraded constituents. As cells age, their ability to perform these functions declines. This likely leads to an unsustainable accumulation of protein aggregates, which ultimately present an obstacle to cellular survival^1–3^. Indeed, studies of the distinct signaling networks in *C. elegans* that modulate lifespan have provided evidence of a central role for autophagy in many known longevity paradigms. These pathways include the highly conserved mechanistic target of rapamycin (mTOR), insulin/IGF-1 like (IIS), and 5′ AMP-activated protein kinase (AMPK) pathways. Notably, the inhibition of autophagy in any model of longevity mediated through the mTOR, IIS, or AMPK nutrient sensing pathways strongly suppresses lifespan^4, 5^. In mammals, global defects in autophagy are lethal postnatally, while tissue-restricted deficiencies produce age-associated pathologic features, including accumulation of inclusion bodies containing ubiquitinylated proteins, deformed mitochondria, ER stress, and appearance of lipofuscin positive vesicles^5^. These local defects in autophagy usually result in organ-specific dysfunction, likely due to the diverse functional roles of autophagy; tissue-restricted autophagy defects have been investigated in some tissues (hepatocytes, neurons, skeletal and cardiac muscle, immune cells)^5^.

Regulation of autophagy by conserved signaling pathways is primarily understood at post-translational levels; relatively few transcriptional regulators that operate broadly downstream of nutrient sensing pathways to regulate autophagy have been identified^6^. How autophagy is transcriptionally regulated under diverse upstream stimuli therefore remains unclear. *Pha-4*/FoxA is required for lifespan extension in the *C. elegans eat-2* mutant model of dietary restriction and regulates autophagy, but is dispensable in other models such as IIS signaling-deficient nematodes or other modes of dietary restriction^7–9^. A recently identified TFEB ortholog, HLH-30, has been shown to attenuate lifespan across multiple *C. elegans* longevity models and to regulate autophagy; it remains to be seen whether TFEB has any direct influence on mammalian aging^6^. In mammals, substantial work has been performed on the transcriptional regulation of autophagy. Among others, β-catenin, C/EBPβ, FOXO1/3, GATA1, HIF1, NF- κB, p53, and SREBP2 have been reported to be regulators of autophagy, primarily through direct transcriptional activation of autophagy genes^10^. Additionally, TFEB, an activator, and ZKSCAN3, a repressor, have been reported to bind directly to promoter regions of target lysosomal and autophagy genes to regulate autophagosome and lysosome biogenesis in an organized pattern of control known as the coordinated lysosomal expression and regulation (CLEAR) network^11, 12^. Their roles in the connection between autophagy and mammalian aging largely remain to be explored, although variants of FOXO3A in humans have been linked to longevity in seven cohorts globally^13^.

The Kruppel-like transcription factors (KLFs) are a subfamily of zinc finger transcriptional regulators with highly characterized roles in proliferation, survival, metabolism, and response to stress. While 18 exist in mammals, in *C. elegans* 3 *klf* encoding genes (*klf-1, klf-2* and *klf-3*) have been identified with roles in fat metabolism, cell survival, and muscle attachment^14, 15^. We and others have provided evidence that mammalian Kruppel-like factors such as KLF4 may have a role in the regulation of autophagy. Using an in vitro model of multiple myeloma, Riz et al.^16^ showed that *KLF4* regulated *SQSTM1* and contributed to carfilzomib resistance. Liu et al.^17^ found that mouse embryonic fibroblasts (MEFs) lacking *Klf4* exhibited impaired autophagy, increased apoptosis, and increased DNA damage, at least partially due to enhanced mTOR signaling. Finally, studies from our laboratory have reported that KLF4 regulation of mitophagy in cardiomyocytes is critical for mitochondrial homeostasis and the cardiac response to stress occurring with pressure overload. Cardiac KLF4 is required for the optimal function of an estrogen-related receptor/PPARγ coactivator 1 module to bind to metabolic and mitochondrial targets^18^. A recent link to lifespan has been established by Carrano et al.^19^ whereby modulation of nematode lifespan via the HECT ubiquitin E3 ligase *wwp-1* is dependent on *klf-1* monoubiquitylation. However, whether KLFs are required for normal lifespan and the mechanistic basis by which they affect lifespan in *C. elegans* remain unknown, as does the existence of an ortholog with effects on mammalian aging.

Here we demonstrate that the KLFs are conserved regulators of autophagy and are necessary and sufficient for lifespan extension in separate longevity paradigms. We find that *C. elegans klf-1* and *klf-3* are broadly required across mechanistically distinct longevity paradigms. Further, from the known mammalian KLFs, we identify KLF4 as a direct transcriptional regulator of autophagy. Finally, while KLF4 has profound pleiotropic effects in multiple cell types, it functions to regulate autophagy in vascular endothelial cells and modulate blood vessel aging.

## Results

### KLF requirement for lifespan extension in multiple pathways

To assess the influence of KLFs on lifespan and determine which KLFs may be involved, we first performed single and combinatorial loss-of-function analysis of the three known *C. elegans* KLFs. Genetic loss of single *klf* genes achieved by either RNA interference (RNAi) by feeding during adulthood or knockout models showed little to no modulatory effect on lifespan (Supplementary Tables 1 and 2). Combinatorial deficiency achieved by knockdown of one *klf* in the background of a second *klf* mutant nematode revealed that knockdown of *klf-2* in both a *klf-1(tm731)* mutant and *klf-3(ok1975)* mutant did not affect lifespan, while the converse, single knockdowns of *klf-1* or *klf-3* in a *klf-2(ok1043)* mutant, reduced mean lifespan significantly, a result which may reflect the differential response of an organism to acute vs. chronic loss of a *klf* gene (Supplementary Fig. 1B, C, E, F; Supplementary Tables 1 and 2). However, double loss of *klf-1* and *klf-3* in any combination displayed a robust reduction in lifespan compared to wild-type nematodes (Supplementary Fig. 1A, D). Together, our findings demonstrate that *klf-1* and *klf-3* are required for normal lifespan in *C. elegans*.

Interestingly, we observed that RNAi feeding targeting *klf-3* from hatching was lethal but *klf-3(ok1975)* mutants were viable. Therefore, we hypothesized that *klf-3(ok1975)* mutants had compensated for *klf-3* loss by induction of *klf-1*. To address this, we measured transcript levels of *klf-1* in worms with single knockdown or knockout of *klf-3* and vice versa. *Klf-1* transcript levels were non-significantly different in *klf-3* knockdown worms compared to wild-type, but were upregulated 1.4-fold in *klf-3(ok1975)* mutants. *Klf-3* transcript levels were also not significantly changed in *klf-1* knockdown worms compared to wild type, but were increased 3.5-fold in *klf-1(tm731)* mutants, providing evidence of compensatory induction of KLF family members in mutant worms but not in RNAi-treated animals. Given the relatively modest upregulation of *klf-1* transcript levels however, it remains unclear whether compensatory induction can be the sole explanation for the apparent lethality of *klf-3* knockdown begun from hatching, or whether other mechanisms may be operative (Supplementary Fig. 2; Supplementary Table 2).

**Fig. 1:**
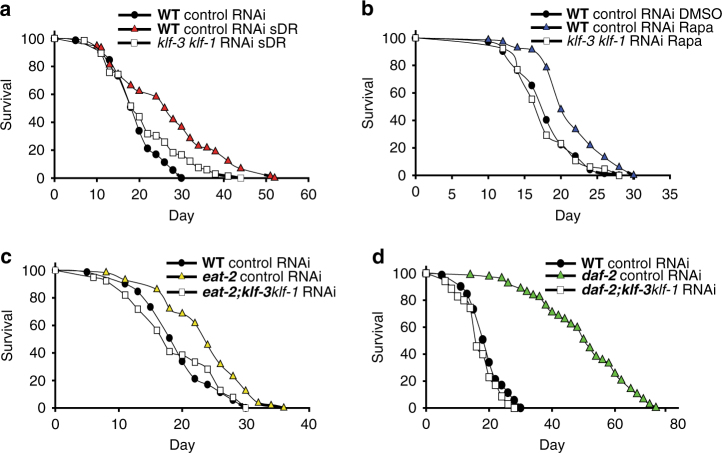
KLFs are required for long lifespan in multiple longevity paradigms. Lifespan analysis of animals subjected to solid agar dietary restriction at 10^8^ cfu/ml OP50 (sDR) (**a**), 100 µM rapamycin (Rapa) (**b**), *eat-2(ad1116)* animals (**c**), and *daf-2*(*e1370*) animals (**d**) after double loss-of-function of *klf-1* and *klf-3*. Animals were crossed into *klf-3(ok1975)* mutant to achieve *klf-3* loss of function, and RNAi feeding targeting *klf-1* was used to achieve *klf-1* loss of function starting from day 1 of adulthood. sDR and rapamycin treatments were initiated from day 1 of adulthood to avoid developmental alterations. All lines were raised and maintained on OP50 at 20 °C. Strain is represented in *bold*. *P*-value < 0.05 by Mantel–Cox log-rank tests. Data are reproduced in Supplementary Fig. 3 with inclusion of *klf-1* loss-of-function groups. See also Supplementary Table 3 for details of lifespan analyses and replicate experiments

We next investigated the potential role of *klf-1* and *klf-3* in mediating lifespan extension in known nutrient sensing longevity pathways. We performed *klf-1*/*klf-3* double loss-of-function experiments in mechanistically separate models of lifespan extension utilizing RNAi against *klf-1* on a *klf-3(ok1975)* mutant background. Strikingly, loss of function of both *klf-1* and *klf-3* abolished lifespan extension in the *eat-2(ad1116)* and food dilution on solid agar (sDR) models of dietary restriction as well as in the *daf-2(e1370)* and rapamycin treated worms, (Fig. 1a–d; Supplementary Table 3) Loss of both *klf-1* and *klf-3* genes was not significantly different from single loss in *eat-2(ad1116)* and animals undergoing sDR, but was additive in *daf-2(e1370)* animals and animals treated with rapamycin, suggesting partial redundancy in *klf-1* and *klf-3* gene targets (Supplementary Fig. 3). The previous finding that single *klf* depletions in wild-type animals did not significantly affect lifespan further points to a specific effect of *klf-1* and *klf-3* on lifespan. Collectively, these results provide evidence for the broad requirement of *klf-1* and *klf-3* transcriptional regulation in lifespan extension mediated through AMPK, TOR, and IIS signaling.

### *klf-3* overexpression enhances health and lifespan in *C. elegans*

**Fig. 2:**
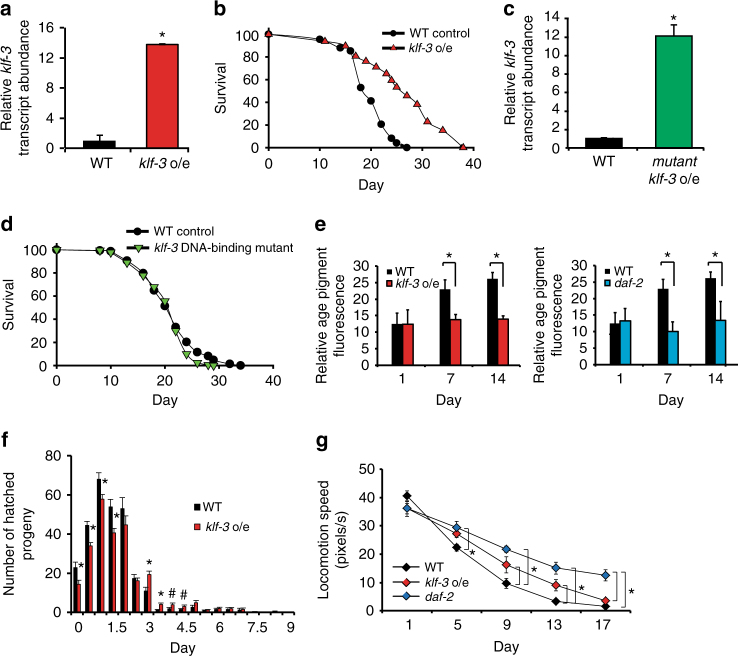
*Klf-3* overexpression extends health and lifespan in *C. elegans*. **a** *klf-3* transcript levels in day 1 wild-type and *klf-3* o/e animals overexpressing *klf-3* driven by putative endogenous promoter as determined by qPCR. Animals were fed OP50 and maintained at 20 °C. Significance determined by Student’s *T*-test, *P-value < 0.05. *N* = 3 biological replicates. **b** Lifespan analysis of *klf-3* o/e animals compared to wild type maintained at 20 °C on OP50. *P*-value < 0.05 by Mantel–Cox log-rank tests. See also Supplementary Table 4 for details of lifespan analyses and replicate experiments. **c** Mutant *klf-3* transcript levels in day 1 animals overexpressing mutated *klf-3* driven by putative endogenous promoter as determined by qPCR. Animals were fed OP50 and maintained at 20 °C. Mutant *klf-3* was created using a 258 nucleotide C-terminal deletion that included the entire zinc-finger-containing region. Significance determined by Student’s *T*-test, *P*-value < 0.05. *N* = 3 biological replicates. **d** Lifespan analysis of *klf-3* mutant overexpressing nematodes grown and maintained at 20 °C on OP50. *P*-value > 0.05 by Mantel–Cox log-rank tests. See also Supplementary Table 4 for details of lifespan analyses and replicate experiments. **e** Appearance of age-related pigments in *klf-3* o/e worms compared to wild-type and *daf-2* mutants compared to wild type as measured by autofluorescence at 420–440 nm (*N* = 10 per group). Data collected from the same wild-type animals are displayed in both panels. Animals were fed OP50 and maintained at 20 °C. Prior to imaging worms were anesthetized by sodium azide solution. Student’s *T*-test, *P-value < 0.05. **f** Reproductive analysis as measured by viable egg-laying by *klf-3 o/e* and wild-type animals (*N* = 10 per group). Worms were transferred every 12 h and viable eggs were counted. Student’s *T*-test, *P-value < 0.05. **g** Age-related decay in locomotory speed in *klf-3* o/e worms compared to wild-type worms and *daf-2* mutants (*N* = 10 per group). Worms were picked onto fresh NGM plate without OP50 and scored for 2 min before being returned to plate. Otherwise, animals were fed OP50 and maintained at 20 °C. See also Supplementary Movies 1–6. *P-value < 0.05 after two-way analysis of variance followed by the Tukey’s post hoc test. All *error bars* represent standard error of the mean (SEM)

To demonstrate that *klf-1* and *klf-3* are not only required but also are sufficient for lifespan extension, we performed gain-of-function analyses. We generated transgenic worms overexpressing *klf-3* (*klf-3 o/e*) driven by a 2.5 kb region upstream of the transcription start site and found that lifespan was extended significantly (Fig. 2a, b; Supplementary Table 4). As reported by Carrano et al.,^19^ overexpression of *klf-1* more modestly extended lifespan, and only when driven by the intestine specific *ges-1* promoter (*klf-1 o/e* worm)^16^. We therefore focused efforts on the more long-lived *klf-3 o/e* animals. Overexpression of *klf-3* with a deletion of the zinc finger DNA-binding region abolished lifespan extension, which may suggest that the *klf-3-*mediated effect on longevity requires direct transcriptional regulation of gene targets (Fig. 2c, d; Supplementary Table 4).

In common with other manipulations found to extend lifespan in nematodes, overexpression of *klf-3* not only extended lifespan, but delayed nematode specific aging characteristics. The appearance of age-associated pigments in the nematode intestine was delayed in *klf-3* o/e worms compared to wild type with *daf-2(e1370)* mutants used as positive control, and *klf-3* o/e worms had an extended reproductive period with 50% laying viable eggs at 6 days compared to 5.7 in wild type with no significant change in total number of hatched eggs (Fig. 2e, f; Supplementary Fig. 4). Additionally, the rate of age-related decline in motor activity, as measured by locomotion speed, was reduced in *klf-3* o/e worms (Fig. 2g; Supplementary Movies 1–6), although not as strongly as in *daf-2(e1370)* mutants. There was no significant difference in pharyngeal pumping rates between wild-type and *klf-3* o/e worms, arguing against a mechanical defect in pumping leading to restricted food intake being responsible for the observed changes (Supplementary Fig. 5). Together, our gain-of-function and loss-of-function analyses, as well as previous analysis by Carrano et al.^19^ utilizing intestinal *klf-1* gain-of-function nematodes, suggest that *klf-1* and *klf-3* in *C. elegans* are required and sufficient for health and longevity.

### KLF-mediated lifespan extension is dependent on regulation of autophagy

**Fig. 3:**
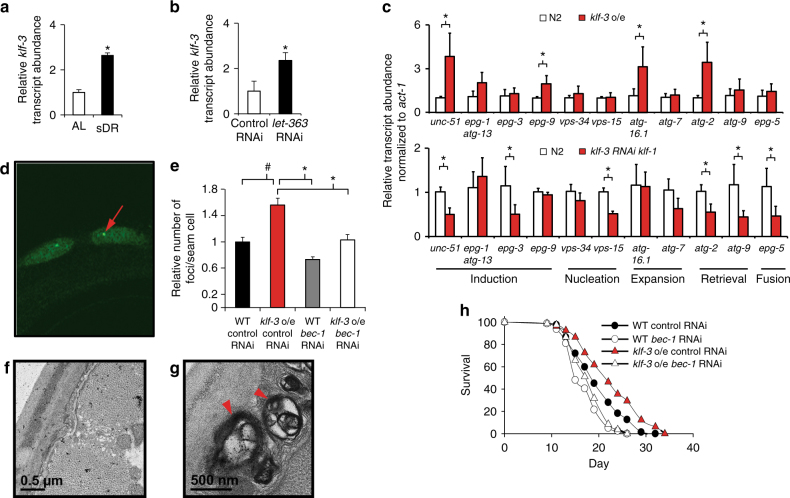
KLF-mediated lifespan extension is dependent on autophagy*. Klf-3* transcript levels in wild-type animals subjected to 2 days of chronic dietary restriction (sDR, OP50 diluted to 10^8^ cfu/ml) (**a**) or inhibition of TOR signaling by RNAi against *let-363* (**b**) starting from day 1 of adulthood. All lines were raised and maintained at 20 °C. *P-value < 0.05 by Student’s *T*-test, *N* = 3 biological replicates. **c** qPCR analysis of a panel of autophagy-related genes in day 1 *klf-3* o/e and loss of function of both *klf-3* and *klf-1* animals compared to wild type. Double loss of function of *klf-3* and *klf-1* was performed as described previously utilizing the *klf-3(ok175)* mutant and simultaneous RNAi feeding targeting *klf-1*. All lines were raised and maintained at 20 °C. *P-value < 0.05 by Student’s *T*-test, *N* = 3 biological replicates (**d**, representative picture). **e** Autophagy in *klf-3* o/e animals as determined by numbers of GFP::LGG-1 punctae in seam cells (*red arrow* denotes GFP-positive puncta) with knockdown of *bec-1* in both wild-type and *klf-3* o/e animals. *P-value < 0.05, ^#^ *P*-value ≤ 0.1 after one-way analysis of variance followed by the Tukey’s post hoc test. *N* = 10–20 animals counted. (**f**, wild-type representative image, **g**, *klf-3* o/e representative image) Electron microscopy images of *klf-3* o/e and wild-type animals in animals aged 9 days. *Arrowheads* indicate sizeable (≥500 nm) autolysosomes as recognized by single-membrane limited vacuolar structures with visible mixed cytoplasmic contents. Full images reproduced in Supplementary Fig. 11, with additional images. **h** Lifespan analysis of wild-type and *klf-3* o/e animals fed RNAi bacteria targeting *bec-1* from day 1 of adulthood. All lines were raised and maintained at 20 °C. *P*-value < 0.05 by Mantel–Cox log-rank tests. See also Supplementary Table 6 for details of lifespan analyses and replicate experiments. All *error bars* represent standard error of the mean (SEM)

The breadth of *klf-1* and *klf-3* influence on lifespan suggested a common mechanism shared among the *daf-2(e1370)*, *eat-2(ad1116)*, sDR, and rapamycin lifespan extending regimens. It has been demonstrated that extended lifespan of these models is dependent on enhanced autophagy, a conserved mechanism critical for cellular homeostasis^20^. Nematodes subjected to sDR or RNAi against *let-363* (*C. elegans* Tor, orthologous to mechanistic target of rapamycin, mTOR) demonstrated induction of *klf-1* and *klf-3* (Fig. 3a, b). This was also observed in the *daf-2(e1370)* and *eat-2(ad1116)* mutant nematodes. (Supplementary Fig. 6). Overexpression of *klf-3* induced the expression of several autophagy-related genes, while loss of function of both *klf-1* and *klf-3* suppressed expression (Fig. 3c), including *unc-51*, *atg-2*, and *epg-2*, suggesting *klf-1* and *klf-3* are positive transcriptional regulators of autophagy gene products. Further, nematodes overexpressing *klf-3* with a deletion of the zinc finger DNA-binding region displayed no significant changes in transcript levels of the same genes (Supplementary Fig. 7).Consistent with this hypothesis, promoter analyses of these autophagy-related genes revealed one or more consensus KLF-binding elements (CA/GCCC) within 1000 bp upstream or 200 base pairs downstream of the transcription start site (Supplementary Table 5). In support of the existence of overlap in autophagy gene targets of *klf-1* and *klf-3*, *klf-3(ok1975)* single mutants demonstrated only modest reduction in transcript levels of the analyzed autophagy-related genes while *klf-3(ok1975)* mutants fed RNAi targeting *klf-1* displayed a significant reduction in the expression of the same autophagy gene targets (Supplementary Fig. 8). Additionally, we independently generated *klf-1 o/e* animals driven by the *ges-1* promoter and found they also displayed enhanced expression of several autophagy-related genes (Supplementary Figs. 9 and 10).

Given these observations, we next conducted an analysis of autophagy. A GFP::LGG-1 (*C. elegans* ortholog of mammalian microtubule-associated protein light chain 3, MAPLC3A and MAPLC3B) translational reporter animal overexpressing *klf-3* demonstrated increased GFP-positive puncta in hypodermal seam cells, an effect weakened by RNAi feeding targeting *bec-1*, while increased autophagic-like vesicles and autolysosomes with heterogeneous intraluminal contents, including electron dense material, were also found in *klf-3* o/e worms by electron microscopy (Fig. 3d–g). Few to no autophagic-like vesicles could be detected by EM in age-matched wild-type or *klf-3* worms fed RNAi targeting *klf-1* (Supplementary Fig. 11). Finally, we sought to determine whether autophagy was required for *klf-3*-mediated lifespan extension. Importantly, the genetic inhibition of autophagy by RNAi against *bec-1*, *atg-13*, *lgg-3*, or *atg-7* either strongly or moderately suppressed lifespan extension of the *klf-3* o/e worms and RNAi against *atg-13* and *bec-1* suppressed lifespan extension in the *klf-1 o/e* worms, findings which remained in line with our gene expression data demonstrating redundant regulation of autophagy-related gene targets by *klf-1* and *klf-3* (Fig. 3h; Supplementary Fig. 12; Supplementary Table 6). Deficiency of *klf-1* and *klf-3* in *daf-2(e1370)* and *eat-2(ad1116)* animals reduced appearance of GFP-positive puncta (Supplementary Fig. 13). Therefore, *klf-3* appears to be a regulator of autophagy in nematodes, and its influence on lifespan is autophagy-dependent.

### Mammalian KLF4 regulates autophagy

**Fig. 4:**
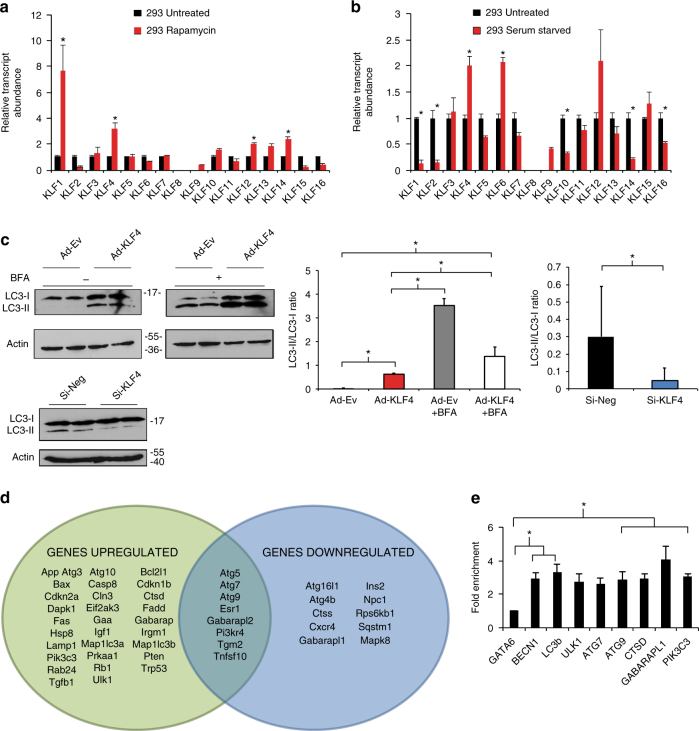
KLF regulation of autophagy is conserved in mammalian cells. qPCR screens of HEK293 cells exposed to rapamycin treatment (**a**) and starvation (**b**). Cells were treated with respective regimen for 2 days prior to RNA isolation and qPCR analysis. Further details are given in Methods section. *P-value < 0.05 by Student’s *T*-test, *N* = 3 biological replicates. **c** Western blot analysis of LC3-I lipidation with and without KLF4 manipulation. Briefly, MEFs were treated with adenoviral *Klf4* or siRNA targeting *Klf4* and effects assessed by western blot 2 days afterwards. Data shown are representative of two independent experiments. Graphs represent quantitated LC3-II/LC3-I ratios. *P-value < 0.05 by Student’s *T*-test or after one-way analysis of variance followed by Tukey’s post hoc test. Further details in Methods section. **d** Autophagy pathway qPCR array analysis in MEFs with Ad-KLF4 or Si-KLF4 normalized to appropriate viral or siRNA control. MEFs were treated for 72 h prior to RNA isolation and qPCR analysis. *Green oval* represents significantly induced genes by Ad-KLF4 and *blue oval* represents genes reduced by Si-KLF4. *P*-value < 0.05 by Student’s *T*-test. *N* = 3 biological replicates. See also Supplementary Table 7 for full gene list of fold changes and *P*-values. **e** ChIP-qPCR in MEFs treated with control or adenoviral KLF4 of several target genes normalized to input DNA, then to nontarget control confirms KLF4 recruitment to CA/GCCC elements in regions upstream of autophagy genes. A locus upstream of GATA6 was used as a nontarget control. *P-value < 0.05 after one-way analysis of variance followed by the Dunnett’s post hoc test. *N* = 3 biological replicates. For list of ChIP-qPCR primers see Supplementary Table 9. All *error bars* represent standard error of the mean (SEM)

To comprehensively investigate a role for the *Klf* gene family in the regulation of autophagy, we performed a qPCR screen for all known mammalian KLFs in HEK293 cells treated with rapamycin or serum starvation, regimens which stimulate autophagy. Although several *Klf* family members were induced by either treatment, *Klf4* was strongly induced by both treatments. These findings were consistent with previous literature (Fig. 4a, b) and encouraged us to pursue studies focused on KLF4^18^. In MEFs, adenoviral overexpression of *Klf4* increased LC3-I lipidation by western blot analysis and the converse by siRNA knockdown was true. To confirm enhanced autophagic flux, we treated MEFs with a late stage autophagy inhibitor, bafilomycin A1 (BFA) a v-ATPase inhibitor which prevents intralysosomal degradation. BFA treatment increased LC3-I lipidation even further, indicating an increase in autophagic flux (Fig. 4c). A qPCR array screening the entire autophagy pathway demonstrated that a large number of genes were up- and downregulated with KLF4 viral manipulation, suggesting a broad effect of KLF4 on the autophagy machinery (Fig. 4d; Supplementary Table 7). Importantly, ChIP-qPCR analysis of KLF4 binding sites (CA/GCCC boxes) on several core autophagy targets provided evidence for increased KLF4 recruitment to target genes (Fig. 4e), suggesting that KLF4 acts on autophagy as a direct regulator as opposed to indirect regulation through cytoplasmic interactions with autophagy-related genes.

### Endothelial restricted KLF4 overexpression delays vessel aging and enhances autophagy

We have found that a *C. elegans* KLF modulates nematode lifespan through regulation of autophagy and that this regulation is functionally conserved via mammalian KLF4. Previous studies by our lab have demonstrated a crucial role for KLF4 in vascular health and function, conferring protection from atherothrombosis, as well as mediating many of the effects of fluid shear stress on the endothelium^21, 22^. In humans, an aged vasculature is the dominant risk factor for the development of cardiovascular disease and is characterized by a number of changes, notably an increase in arterial stiffness and reduction in endothelial-dependent dilation in response to blood flow^23^. We therefore questioned whether vascular KLF4 might also influence mammalian vascular aging.

**Fig. 5:**
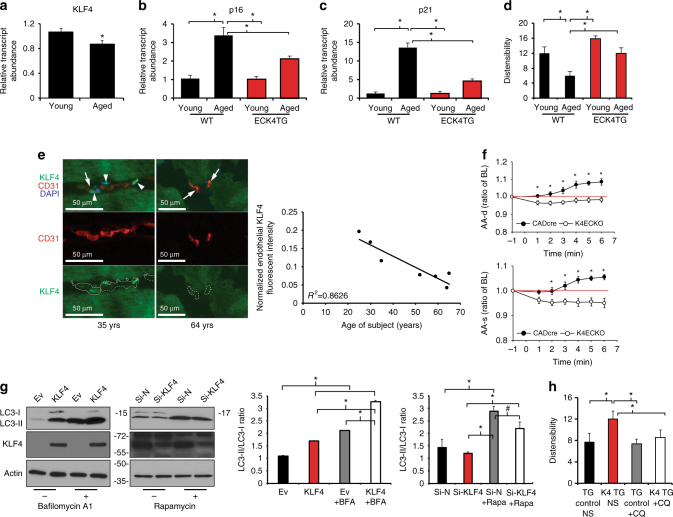
KLF4 regulates autophagy and aging in vasculature and decreases with age. **a** qPCR analysis of *Klf4* in isolated cardiac endothelial cells in young and middle-aged wild-type mice. (Young = 3 months, middle-aged = 10–12 months, *N* = 3 biological replicates). *P-value < 0.05 by Student’s *T*-test. qPCR analysis of *p16* (**b**) and *p21* (**c**) in isolated cardiac endothelial cells in young and aged wild-type and ECK4TG mice (young = 3 months, aged = 10–12 months, *N* = 3 biological replicates). *P-value < 0.05 after one-way analysis of variance followed by the Tukey’s post hoc test. **d** Ascending aorta dilation at baseline in young and aged transgenic control and ECK4TG mice (young = 3 months, aged = 10 months, *N* = 6–9). *P-value < 0.05 after one-way analysis of variance followed by the Tukey’s post hoc test. **e** Expression of CD31 and KLF4 by immunofluorescence, with DAPI staining, representative images. CD31-positive areas are marked by *dotted lines*. *Arrows* indicate CD31-positive endothelial cells. *Arrowheads* indicate KLF4-positive endothelial nuclei. *Scale bar* = 50 μm. Correlation studies were performed with *R* ^2^ = 0.8626 (Pearson correlation, *P*-value = 0.003; *N* = 7 patients). Further details are given in Methods section. **f** Ascending aorta diastolic (AA-d) and systolic (AA-s) diameter in control (CADcre, *N* = 6) and *Klf4* endothelial knockout (K4ECKO, *N* = 9) mice before and during acetylcholine infusion (*t* = 0). Diameter assessed by M-mode echocardiography. Ratio of baseline (Ratio of BL). *P-value < 0.05 by Student’s *T*-test. Further details are given in Methods section. **g** Western analysis of HUVECs overexpressing KLF4 with and without BFA treatment with knockdown of KLF4 in rapamycin treated HUVECs. Briefly, HUVECs were treated with adenoviral KLF4 or siRNA targeting *KLF4* and effects assessed by western blot 2 days afterwards. Data shown are representative of two independent experiments. Graphs represent quantitated LC3-II/LC3-I ratios. *P-value < 0.05, ^#^ *P*-value < 0.1 after one-way analysis of variance followed by Tukey’s post hoc test. Further details are given in Methods section. **h** Administration of chloroquine in middle-aged mice to inhibit autophagy and measurement of ascending aorta dilation in middle-aged transgenic control and ECK4TG mice (aged = 10–12 months, NS = normal saline, CQ = chloroquine, *N* = 6–9). *P-value < 0.05, after one-way analysis of variance followed by the Tukey’s post hoc test. All *error bars* represent standard error of the mean (SEM)

Consistent with a role for KLF4 in mammalian vascular aging, *Klf4* expression decreased with age in isolated murine endothelial cells (Fig. 5a). Indeed, expression of *p16* ^*INK4a*^ */Rb* and *p21*, markers of cellular senescence, was increased with age in isolated endothelial cells from young (2 months) and middle-aged (~ 11 months) animals, and this increase was attenuated in age-matched endothelial restricted *Klf4* transgenic mice (ECK4TG) (Fig. 5b, c)^24^. The physiologic effects of aging on the mammalian arterial tree have long been recognized as an increase in vascular stiffness, an effect ameliorated by the application of caloric restriction^25, 26^. Therefore, we used ECK4TG mice and assessed vascular stiffness in vivo. Vascular distensibility decreased with age in ascending aortas of wild-type mice (Fig. 5d) and its loss was delayed in middle-aged transgenic mice compared with age-matched controls. Finally, we asked whether *KLF4* levels in humans were altered with age. To investigate this, we analyzed skeletal muscle samples obtained from young and old healthy patients. As expected, total VO_2max_ declined with age (Supplementary Fig. 14A). Interestingly, we detected a concurrent age-associated decrease in *KLF4* mRNA transcript levels from RNA isolated from whole samples, suggesting that an age-associated reduction in *KLF4* may have physiologic significance (Supplementary Fig. 14B). Co-staining of skeletal muscle using antibodies directed against KLF4 and CD31 to identify endothelial KLF4 in the microvasculature revealed that levels of vascular KLF4 were strongly decreased with age, localizing a portion of the observed decrease in KLF4 in whole samples to the vascular component (Fig. 5e). Together, these findings point towards a role for vascular KLF4 in mammalian aging.

We further investigated other age-related alterations in the vessel that might contribute to vessel stiffness. Vessel distensibility in vivo may be influenced by structural components as well as functional responses. We observed no significant change in aortic diameter, aortic wall thickness, or aortic wall area between middle-aged ECK4TG and wild-type mice (Supplementary Fig. 15). Quantitative analysis of Trichrome stained sections of murine thoracic aortae revealed little change in intermuscular collagen deposition between aged control and ECK4TG mice (Supplementary Fig. 15). Elastin staining demonstrated no significant differences between wild-type and ECK4TG mice in elastin disorganization and number of breaks (Supplementary Fig. 15). Additionally, aortae demonstrated similar SMC α-actin immunoreactivity with uniform expression, which, together with the unaltered wall thickness, suggested little smooth muscle proliferation or migration (Supplementary Fig. 15). Together, these findings suggest that structural composition of the vessel wall is not a major determinant of the KLF4 effect on vessel distensibility. Next, we sought a functional contribution to distensibility by performing in vivo acetylcholine infusion in wild-type and endothelial-specific *Klf4* knockout mice. The vasodilatory response elicited by acetylcholine is strictly dependent on the endothelium, and the loss of endothelial-specific KLF4 completely abolished this response, suggesting that the effect of KLF4 on vascular distensibility is primarily through its effects in the endothelium (Fig. 5f).

While autophagy has been found to be critical in reducing endothelial lipid burden and maintaining hemostasis after vessel injury, to date, the transcriptional regulation of autophagy in endothelial cells is not well understood^27, 28^. Given our previous findings, we hypothesized that KLF4 modulation of vessel aging might be dependent on regulation of autophagy. We treated human umbilical vein endothelial cells (HUVECs) by serum starvation or rapamycin and found strong induction of *KLF4* (Supplementary Fig. 16). We also found that overexpression of *KLF4* in HUVECs increased LC3-I lipidation. Upon BFA treatment, LC3-I lipidation was more strongly increased, suggesting enhanced autophagic flux, while conversely, siRNA knockdown of *KLF4* weakened autophagy induction by rapamycin treatment (Fig. 5g). A cardinal feature of endothelial function, which decays with age, is the regulation of vascular tone, achieved largely through the generation of nitric oxide and the elevation of expression of endothelial nitric oxide synthase (eNOS) under conditions of laminar blood flow. Importantly, in HUVECs subjected to laminar shear stress, a stimulus that strongly induces *KLF4*, in the presence or absence of autophagy blockade via *ATG7* siRNA knockdown, the increase in eNOS protein levels was blunted, suggesting that regulation of endothelial function is partially autophagy-dependent (Supplementary Fig. 17). Additionally, the improvement of vessel distensibility in middle-aged ECK4TG mice was strongly attenuated upon administration of the autophagy inhibitor chloroquine (Fig. 5h). Collectively, our findings point toward a role for KLF4 in mammalian vascular aging, likely through conserved effects on endothelial autophagy.

## Discussion

Here we demonstrate that the KLFs are critical determinants of aging, influencing both lifespan and age-related deterioration, and are broadly required for lifespan extension in all four mechanistically distinct longevity models tested. Importantly, we provide evidence that these effects are mediated by KLF regulation of autophagy and are conserved in a mammalian system via a functional ortholog, KLF4. Together, our findings provide a role for the KLFs as a transcriptional regulatory point in longevity.

Mechanistically distinct long-lived model organisms share several hallmark features. For example, autophagy is enhanced in models of reduced IIS signaling, TOR inhibition, and dietary restriction^29^. Recent studies have begun to provide insight into how complex and disparate signaling pathways can have coordinated anti-aging responses. For example, the nuclear hormone receptor NHR-62, FOXA ortholog PHA-4, and TFEB ortholog HLH-30 have been found to induce expression of autophagy genes in *C. elegans* and are required for lifespan extension across a variety of dietary restriction models^30^. In addition to these observations, we now demonstrate that the KLFs are required for autophagy under several distinct longevity pathways. We therefore suggest a model by which the transcriptional regulation of autophagy, which must maintain organelle and protein homeostasis in nearly every long-lived state, summates multiple upstream inputs through increased activity of common nodal transcriptional regulators. This may occur through increased KLF activity, and may also involve the previously identified *hlh-30*. This allows unified pro-longevity responses in the face of diverse environmental and nutritional stimuli and implies the existence of convergence points in pathways modulating lifespan which may offer attractive targets for therapeutic intervention. What upstream signals interact with the KLFs and the relationship of the KLFs with other known autophagy transcriptional regulators like *pha-4*/FoxA and *hlh-30* will be important future directions for investigation^29^.

Our genetic loss-of-function data suggest functional redundancy exists between the KLF family members in *C. elegans*. Loss of both *klf-1* and *klf-3* reduce lifespan while single overexpression of either *klf-1* or *klf-3* is sufficient to extend it^19^. Although it is possible that *klf-1* and *klf-3* operate through two independent pathways to modulate lifespan, our qPCR analysis demonstrating stronger reduction in transcript levels of autophagy genes with *klf-1* and *klf-3* loss compared to single loss argues more strongly for shared gene targets between *klf-1* and *klf-3*. Such robustness in regulation of critical processes such as autophagy is not surprising and is likely not unique to the KLF family of transcriptional regulators, which may have implications for the discovery of novel longevity related genes through high-throughput screens.

Our studies are consistent with the body of evidence supporting the positive role of autophagy in health and longevity across phylogeny. Further our studies add to a growing appreciation of the importance of transcriptional regulation of autophagy in aging. Specifically, we demonstrate that the KLFs are direct transcriptional regulators of autophagy, likely through direct regulation of a broad array of genes with distinct functions in the autophagic process. This regulation may be particularly relevant in defending against the repeated, chronic insults that occur during aging.

In *C. elegans*, *klf-1* has been implicated in fat regulation and apoptosis, while *klf-3* regulates lipid transport and metabolism^14, 15, 29^. We report here that the KLFs regulate autophagy as well and that their effects on lifespan are dependent on it. However, our data do not exclude the contribution of other KLF-regulated pathways such as fat metabolism; indeed, it is likely that autophagy may be involved in or mediate the effects of lipid metabolism on lifespan^31^. The molecular mechanisms linking lipid metabolism and autophagy and how they impact health and longevity remain to be elucidated^32, 33^.

The identification of a functional analog, KLF4, in a mammalian system raises the possibility that its modulatory effects on longevity may also be conserved as well. In humans, cardiovascular disease remains the leading cause of death in developed countries. Accumulating evidence suggest that dysfunction of the vascular endothelium underlies a number of cardiovascular diseases of aging including hypertension (vessel tone), atherosclerosis, and calcification. *Klf4* is expressed in a diverse set of mammalian tissues, including endothelial cells and our findings point to KLF4 as serving an essential role in vascular health and aging^34^. In rat carotid arteries, *Klf4* is induced by rapamycin, while we previously demonstrated endothelial KLF4 to be protective against atherothrombosis, to generate an anti-inflammatory endothelial phenotype, and to be required for mitochondrial turnover in cardiac muscle cells^18, 35–37^. Here we show additionally that endothelial-specific manipulation of KLF4 modulates several characteristic features of vessel aging, namely a decline in eNOS expression, increased stiffness, and a rise in endothelial replicative senescence. Combined with prior observations on the requirement of KLF4 for mechanotransduction pathways in endothelial cells, we propose that age-associated increases in disturbed blood flow lead to lowered levels of KLF4 in blood vessels to promote endothelial dysfunction and, over time, contribute to the increased risk of atherosclerosis and other vessel-related pathology seen in aged individuals^38, 39^. Our findings provide a novel physiological context for KLF4 in maintaining healthy, youthful endothelium whereby the beneficial effect of laminar shear stress on endothelium may be dependent on KLF4 regulation of autophagy. Our results also support the notion that endothelial autophagy may be sensitive to mechanical conditions created by blood flow in addition to nutrient status, which likely reflects the specialized nature of many endothelial functions.

Notably, the regulation of autophagy in the endothelium requires further study and, in recent years, this topic has attracted growing interest. The health of the endothelium is essential to its functions at the interface of circulating fluid and the vessel wall. As an integrator and transducer of various physiologic stimuli, the endothelium is involved in many processes including the maintenance of a semi-permeable barrier, blood fluidity, and vasoreactivity. Dysfunction of the endothelium also plays a critical role in the development of vascular pathology such as atherosclerosis. An accumulating body of evidence suggests diminished endothelial autophagy may underlie endothelial dysfunction stemming from a diverse set of risk factors. For example, in a series of studies, Torisu et al.^27, 28^ provided evidence that mice with endothelial-specific loss of *Atg5* and *Atg7* exhibit impaired secretion of a major regulator of blood fluidity, von Willebrand factor homolog, suggesting that reduced autophagy in the endothelium may contribute to hypercoagulable states. On the other hand, increased autophagy in endothelial cells is protective against harmful effects of oxidized low-density lipoprotein, reactive oxygen species, hypoxia, and advanced glycation end products, observations in line with the finding that treatment of atherosusceptible (LDLR−/−) mice with an mTOR inhibitor was protective^40–45^. Finally, attenuated autophagy is correlated with reduced arterial endothelium-dependent dilatation in aortas of old mice and treatment with trehalose to induce autophagy rescues the reduction^46^. The precise mechanisms by which endothelial autophagy mediates these effects, mechanisms that include alterations in NO bioavailability, oxidative stress, and inflammation, remain active areas of investigation.

Finally, the degree in which the endothelial organ contributes to organismal aging remains an open and complex question, as does the question of whether KLF4 in other tissues has roles in mammalian aging as well. The ubiquitous nature of the endothelium almost certainly necessitates context specific interactions with the surrounding tissue. The marked decrease in vascular KLF4 in skeletal muscle we observe here likely has detrimental effects on muscle function; indeed, the role of small vessel function (e.g. blood flow, angiogenic capacity) in exercise adaptation is well known^47, 48^. The age-associated alterations we find in VO_2max_ therefore are likely a consequence of alterations both in skeletal as well as vascular components. Additionally, our data do not exclude some contribution of skeletal muscle KLF4 to age-related change. Tissue-restricted and inducible gene expression studies may offer additional insights into the spatial and temporal contribution of the KLFs to longevity, and provide further avenues of investigation.

## Methods

### Strains, maintenance, and preparation

N2 Bristol was used as the wild-type strain. These mutant strains were obtained from Caenorhabditis Genetics Center (CGC, University of Minnesota, Minneapolis, MN, USA): *klf-1(tm731), klf-2(ok1043), klf-3(ok1975), daf-2 (e1370), eat-2(ad1116)*. For GFP::LGG-1 puncta autophagy experiments, adIs2122 Ex [*lgg-1*p::GFP::*lgg-1* + *rol-6*(su1006)] was used. Worm culture on nematode growth medium (NGM) and age synchronization performed according to standard methods^49^. All strains were out-crossed at least three times with N2 nematodes.

### Generation of transgenic and mutant *C. elegans* lines

Transgenic worms overexpressing *klf-3* or *klf-3* zinc finger mutant were generated as described^50^. Specifically, *klf-3* genomic DNA was under the control of putative native promoter (~2.5 kb upstream of start codon) and the fluorescent marker Discosoma sp. red fluorescent protein (DsRed) was under the control of the promoter of *myo-3*. For intestinal restricted nematodes overexpressing *klf-1, klf-1* genomic DNA was under the control of a ~3 kb region upstream of *ges-1*, and assembled by Gibson assembly and sequenced. For nematodes overexpressing mutant *klf-3*, a 258 nucleotide C-terminal deletion that included the entire zinc-finger-containing region was generated using the QuikChange II Site-Directed Mutagenesis Kit. Double mutant *daf-2*;*klf-3* and *eat-2*;*klf-3* were generated by crossing hermaphrodite *daf-2* or *eat-2* animals to *klf-3* males and genotyped by PCR or by placing worms at 25 °C, at which *daf-2* mutants enter the dauer stage. Primer sequences used were 5′-gcaaaagaggatgggaatca-3′ (forward primer outside of deleted region), 5′-gtaggtggtctagtaccact-3′ (forward primer within deleted region), 5′-aaagcaaaaatgacatcgcc-3′ (reverse primer) for *klf-3* and 5′-ctctagagttggctaaccttc3′, 5′gaacgcttgcaaattcgctgc-3′ for *eat-2*.

### RNAi clones

RNAi clones (from Julie Ahringer's RNAi library) sequenced using M13-forward primer to verify identity. RNAi bacteria (HT115) containing vectors expressing dsRNA targeting gene of interest were grown in LB at 37 °C containing 10 μg/ml tetracycline and 50 μg/ml carbenicillin, induced with 1mM IPTG, and then seeded onto NGM-carbenicillin plates.

### Dietary restriction treatments

Solid agar dietary restriction (sDR) in *C. elegans*, a particular form of food restriction requiring the action of AMPK, was performed^51^. Briefly, adult worms were transferred every 2 days onto freshly seeded plates at 10^8^ cfu/ml at day 5 of adulthood and lifespan measured. 100 μg/ml carbenicillin and 50 μg/ml kanamycin was applied to bacteria prior to seeding on plates to arrest growth. For cell culture experiments, starvation was accomplished via replacement of media with DPBS for the 1 h before harvest.

### Rapamycin treatments

Rapamycin (LC laboratories) treatment in *C. elegans* was carried out by dissolving rapamycin in DMSO to 50 mg/ml and adding to agar plates to 100 μM concentration^52^. Control plates contained equal concentration of DMSO only. Rapamycin treatment in cell culture was performed by dissolving rapamycin in DMSO and adding to cell culture media to 20 μg/ml. Cells were incubated for 24 h before harvest.

### Lifespan analysis

Lifespan assays were carried out at 20 °C as described^53, 54^. Animals were fed OP50 on NGM plates at 20 °C for at least two generations prior to lifespan analysis. For RNAi experiments, synchronized eggs were added to plates with control bacteria (containing empty vector L4440) then transferred to plates with target RNAi bacteria approximately 3 days later (L4/Young Adult). Nematodes were transferred to plates containing fresh RNAi bacteria every 2 days until the end of egg-laying, then transferred every 5–7 days for rest of lifespan experiment. Nematode viability was scored every 2–3 days. For lifespan analyses, pre-fertile period of adulthood was defined as *t* = 0. Per strain, animal number per assay ranged from 60–180, while valid animal number ranged from 40 to 170. Mean life was defined as the day at which 50% of the population was dead. Log-rank (Mantel-Cox) test was used for all lifespan analyses.

### Autofluorescence quantification

Experiments were performed with a TCS SP2 confocal microscope (Leica Microsystems, Bannockburn, IL, USA) as described^55^. Wavelengths used were 420–440 nm (*λ*_em_).

### Reproduction analysis

Reproduction was analyzed as described with modification^56^. Progeny was counted every 12 h instead of daily.

### Pharyngeal pumping

Synchronized worms were placed on plates in the presence of OP50, left overnight, and the number of pumps (as determined by number of backward grinder movements in the terminal bulb) was counted for 15–30 s (or the length of time for 60 grinder movements), then converted to pumps per minute. At least 10 animals per group were measured and averaged.

### Transmission electron microscopy

Whole nematode animals were picked into triple aldehyde-DMSO, then sequentially exposed to ferrocyanide-reduced osmium tetroxide and acidified uranyl acetate. Samples were dehydrated in ascending concentrations of ethanol and passed through propylene oxide before being embedded in Poly/Bed resin (Polysciences Inc., 21844-1). Acidified uranyl acetate was used to stain thin sections and a modification of Sato's triple lead stain was used before examination using a JEOL 1200EX electron microscope^57^. All EM images were independently analyzed by an EM expert from the Electron Microscopy Facility, Case Western Reserve University, and autophagic vesicles were identified as described by Zhang et al.^58^.

### GFP-LGG-1 puncta quantification

Autophagy was assessed using a GFP::LGG-1 translational reporter characterized previously^59^. GFP-positive puncta were counted in 30–150 total hypodermal seam cells in 10–20 animals, averaged, and analyzed by two-tailed Student’s *T*-test.

### Quantification of locomotion activity

An automated worm-tracking system developed by the lab was used to assay locomotory behavior of worms on NGM plates throughout lifespan as described previously^60, 61^. The worm-tracking system includes a stereomicroscope (Zeiss Stemi 2000C) mounted with a digital camera (Cohu 7800) and a digital motion system (Parker Automation). Using a customer-developed software package, worm images were recorded at 2Hz for 2 min total, and mean centroid speed of the nematode was quantified in real time. Vision data was recorded, compressed and saved as AVI files, and first 30 seconds discarded. *P*-values were generated by two-way ANOVA with the Tukey’s post hoc using Sigmaplot 12 software.

### EC isolation

Cardiac microvascular endothelial cells were isolated from the heart tissue of mice using a standard technique as previously described with a minor modification^62^. Briefly, hearts were washed in ice-cold PBS, minced with razor blades and digested in 1% BSA, collagenase type I, 1 mM CaCl_2_ and 1mM MgCl_2_ in PBS at 37 °C for 45 min. DynaI bead-conjugated anti-PECAM antibody was used to purify endothelial cells (BD Biosciences, San Jose, CA, USA). ECs were pooled from six mice (two mice per isolation for biological triplicate), and immediately harvested for fresh RNA.

### RNA extraction and qPCR

*C. elegans* samples were homogenized in TRIzol reagent (Life Technologies, 15596-026) with a TissueLyser (Qiagen) or subjected to at least three freeze–thaw cycles. Cell samples were directly dissolved in TRIzol reagent. The High Pure RNA isolation kit (Roche, 11828665001) or Aurum Total RNA Fatty and Fibrous Tissue Kit (Bio-Rad, 732-6830) were used to isolate total RNA, which was subsequently treated with DNase I (Life Technologies, 18068015). iScript Reverse Transcription Kit (Bio-Rad, 170-8841) was used to synthesize complementary DNA. Using a ViiA 7 Real-Time PCR System (Applied Biosystems), Taqman (Roche Universal ProbeLibrary System) or SYBR green methods were utilized for qPCR analysis. ΔΔCT method was used to calculate relative expression normalized to mammalian GAPDH or *C. elegans act-1*. PCR primer sequences are listed in Supplementary Table 8, or primers from the RT^2^. Profiler PCR Array Mouse Autophagy (Qiagen) were used.

### Western blot analysis

RIPA buffer (Sigma-Aldrich, R0278) was used for protein extraction. RIPA was supplemented with proteinase/phosphatase inhibitor cocktail (Roche, 4693132001 and 4906845001). LC3B (Cell Signaling, 3868, 1:1000) was used for LC3-I lipidation assays. KLF4 (Santa Cruz (H180) SC-20691, 1:2000), eNOS (BD Biosciences, 610297, 1:2000) were used for other western analyses. Bio-Rad Quantity One software was used for quantitation of LC3-II/LC3-I ratios. Full scans of the western blots are supplied in Supplementary Fig. 18.

### Cell culture and viral infection

HUVECs (Lonza) and HEK293 cells (ATCC CRL-1573) were cultured as described previously^36^. All lines were authenticated by respective sources and tested for mycoplasma. Expression plasmid (KLF4) and adenoviral KLF4 have been described previously^36^. qPCR of total RNA isolated from cell source was used to confirm efficacy of infection. The adenovirus contains human KLF4 (Ad-KLF4). In overexpression experiments, target gene expression was assessed 2 days after infection, with viral constructs without target gene used as controls. Dharmacon On-Target siRNA plus (J-005089-09, 9314; si-KLF4) and non-targeting siRNA control (D-001810-01-05; si-N/si-Neg) were used for KLF4 knockdown experiments. Target gene expression was assessed 2 days after transfection. Si-Atg7 (Thermo Fisher, 135754) was used for *Atg7* knockdown.

### Chromatin immunoprecipitation

1% formaldehyde was used to fix 2 × 10^7^ MEFs. Chromatin was extracted then sonicated via BioRuptor (Diagnode), performed in triplicate. Immunoprecipitation was performed with 2-5 μg of anti-KLF4 antibodies (Santa Cruz, sc-20691) pre-incubated with Protein A/G beads (EMD Millipore, 16-663), with several cycles of washes and elution. Chromatin was reverse cross-linked and genomic DNA purified. qPCR was used to amplify DNA in precipitated and input samples. Nontarget control was a locus upstream of GATA6. Primer sequences used are listed in Supplementary Table 9.

### Chloroquine treatment

Mice were injected once daily (30 mg/kg i.p.) with chloroquine (Sigma) in saline for 8 days. Mice were assessed by echocardiography at baseline prior to initiation of chloroquine treatment, and same mice were assessed again after treatment.

### Acetylcholine infusion

CADcre (VE-cadherin-driven Cre mice backcrossed into the C57BL/6 background, *N* = 6) and ECK4KO (floxed *Klf4* mated to CADcre, *N* = 9), male, 5–7 months old after bone marrow transplantation for 8 weeks were used for this experiment. Mice were anesthetized with Avertin (0.25 mg/g, i.p.) and restricted on a temperature-controlled small animal table to maintain physiological body temperature during experiment, a jugular vein catheter was used for continuous acetylcholine infusion. A Vevo 770 High-Resolution Imaging System equipped with an RMV-707B 30-MHz probe (VisualSonics) was used for echocardiography. M-mode sampling was used through the upper abdominal aorta long axis, images were recorded under baseline and then after continuous acetylcholine infusion (2 μg/kg/min, 10 μl/min, Sigma-Aldrich, St Louis, MO) for the indicated time intervals. The abdominal inner diameter at end-systolic and end-diastolic were analyzed through abdominal aorta M-mode image.

### Immunostaining and histologic and morphometric analysis

Perfusion-fixed aortas were embedded in paraffin and cut into 7-μm-thick sections. Images of whole aorta were taken at ×100 magnification and at ×400 magnification. Medial thickness, lumen diameter, and area were quantified using Image-Pro Plus software (Media Cybernetics). Trichrome staining of tissue was performed using the Gomori’s Trichrome Stain kit (Thermo Scientific) according to the manufacturer’s instructions. Collagen deposition was quantified using Image-Pro Plus software by recognizing area stained blue vs. total area of interest (intermuscular area/total area × 100). For each vessel, 9–10 images at ×400 magnification were quantified. Elastin Stain kit (Sigma) was used to perform staining on perfusion-fixed aortic cross-sections. Each data point represents total number of elastin breaks in a whole vessel cross-section, “elastin break” representing a distinct two-ended discontinuity in a single concentric lamellar ring of elastin. The same aortic levels were used to compare aortic sections from each mouse. Two independent and blinded observers performed measurements. Elastin-stained aortic sections were used to calculate vessel diameter. Lumen diameter was defined as mean distance between internal elastic laminae on opposing sides of the vessel. α-smooth muscle actin staining was performed using α-smooth muscle actin Cy3 (C6198) antibody from Sigma-Aldrich and 4′,6-diamidino-2-phenylindole (DAPI) counterstaining. Cryosections (10 μm thick) were received from the Cleveland Clinic Histology Core. Muscle was encased in tragacanth gum and flash frozen in liquid nitrogen-cooled isopentane. Samples were stored at −80 °C until analysis. Samples fixed in 10% neutral buffered formalin were blocked with 5% normal goat serum and 2% BSA before incubation with primary antibodies for KLF4 (Santa Cruz, SC-20691) and CD31 (BD Pharmingen, 550389) diluted 1:100 in blocking buffer overnight at 4 °C. 1:100 dilution was chosen after CD31 titration to avoid saturation conditions. Secondary Alexa fluor-488 goat anti-rabbit (Life Technologies, A11008), and -594 goat anti-mouse (Invitrogen, A11032) diluted 1:200 in blocking buffer were incubated for 2 h at room temperature. Following treatment with TrueBlack Lipofuscin Autofluorescence Quencher (Biotium, 23007), slides were mounted with mounting medium for fluorescence with DAPI (vector H-1200). Three complete sections were evaluated for each subject. Images covering the entire area of each section were collected and analyzed by a blinded researcher. Images were captured with Leica DMI 6000 B and analyzed using image J. Endothelial area was defined by CD31 positivity. KLF4 levels in those regions was then assessed by fluorescent intensity and normalized to CD31 intensity; we did not find CD31 intensity to vary significantly between different biopsies.

### In vitro hemodynamic flow model

To generate fluid shear stress, a cone and plate viscometer were used as described previously^63^. Briefly, the cone was placed into the 60-mm dish which contains a confluent monolayer of endothelial cells in culture media. Cells were either exposed to laminar flow (shear stress of 17 dyne/cm^2^) or maintained under static condition for 24 h at 37 °C in a 5% CO_2_ incubator. After siRNA knockdown of *Atg7*, protein was isolated after 24 h of flow, at which time a steady-state flow-dependent phenotype emerges.

### Mouse models

All animal studies were carried out with permission, and in accordance with, animal care guidelines from the Institutional Animal Care Use Committee (IACUC) at Case Western Reserve University. Floxed *Klf4* mice (gift from K. Kaestner, University of Pennsylvania, Philadelphia, PA, USA) were mated with VE-cadherin-driven Cre mice to obtain ECK4KO mice. Endothelial specific KLF4 transgenic mice were generated by our laboratory with VE-cadherin promoter (gift from K. Walsh, Boston University, Boston, MA, USA) controlling human KLF4 coding sequence. All mice used were on a C57BL/6J background. Facility housing mice was temperature and humidity controlled, specific pathogen-free. Mice had ad libitum access to water and laboratory rodent chow, and were exposed to a 12 hour light/dark cycle.

### Vascular distensibility and echocardiography

Mice were anesthetized with Avertin (0.25 mg/g i.p.), and transthoracic echocardiography was performed using a Vevo 770 High-Resolution Imaging System equipped with an RMV-707B 30-MHz probe (VisualSonics). Standard M-mode sampling was used at the ascending aorta, and artery diameter at systole and diastole determined using the system software. Distensibility was calculated using the following formula: distensibility = (systolic diameter−diastolic diameter)/diastolic diameter × 100%. Control (C57BL/6 J) at 3 and 10–14 months old and EC-KLF4 Tg male mice at 3 and 10–12 months old were used for vessel compliance analysis by echocardiogram of ascending aorta. Experiments were performed in a nonblinded manner.

### Participants

Ten sedentary individuals (five old, 50–70 year vs. five young 20–40 year) were included in this cross-sectional study. Subjects were non-smoking, weight stable (<2 kg in previous 6 months), and had no chronic diseases (i.e. renal, hepatic, thyroid, cardiovascular). Subjects had not participated in any regular exercise (<30 min of aerobic activity > 2 days/week) for at least 6 months prior to providing the muscle biopsy sample, and all subjects reported similar activity levels via a physical activity questionnaire. All participants were verbally briefed about the study and signed informed consent documents approved by the Institutional Review Board.

Each participant performed an incremental-graded treadmill exercise test to determine maximal oxygen consumption (VO_2max_). Speed was set between 2 and 5 miles/h, and the incline of the treadmill increased 2–3% every 2 min until volitional fatigue. Inspired air volumes were measured from pressure changes detected by a bidirectional digital volume sensor (Triple V) pneumotach, and concentrations of O2 (electrochemical detection) and CO2 (thermal conductivity detection) were measured using a Jaeger OxyCon Pro/Delta System (Version 4.6, Hoechberg, Germany). At least two of the following criteria were required for a maximum test: plateau in VO2, heart rate (HR) within 10 beats/min of age-predicted maximum, and/or a respiratory exchange ratio >1.1.

### Muscle biopsy

Study participants reported to the Clinical Research Unit after an overnight fast and rested for 1 h. Skeletal muscle biopsies were obtained from the vastus lateralis using a Bergstrom needle as previously described^64^. The muscle was flash frozen and stored in liquid nitrogen until processing.

### Statistics

Statistical significance was analyzed using SigmaPlot software. One-tailed unpaired Student’s *t*-tests, one-way or two-way ANOVA, and Mantel–Cox log-rank tests were used for their appropriate applications as indicated in the figure legends. For mouse studies, data passed normality test, and power necessary for effect size based on previous experiments. Student’s *T*-test was used for difference between individual groups and log-rank tests for lifespan studies. In all figures, error bars represent standard error of mean (SEM).

### Data availability

The data sets generated during and/or analyzed during the current study are available from the corresponding author on reasonable request.

## Electronic supplementary material

Supplementary Information

Description of Additional Supplementary Files

Supplementary Movie 1

Supplementary Movie 2

Supplementary Movie 3

Supplementary Movie 4

Supplementary Movie 5

Supplementary Movie 6

## References

[1] Ben-Zvi A, Miller EA, Morimoto RI (2009). Collapse of proteostasis represents an early molecular event in *Caenorhabditis elegans* aging. Proc. Natl Acad. Sci. USA.

[2] Olzscha H (2011). Amyloid-like aggregates sequester numerous metastable proteins with essential cellular functions. Cell.

[3] Woerner AC (2016). Cytoplasmic protein aggregates interfere with nucleocytoplasmic transport of protein and RNA. Science.

[4] Fontana L, Partridge L (2015). Promoting health and longevity through diet: from model organisms to humans. Cell.

[5] Rubinsztein DC, Mariño G, Kroemer G (2011). Autophagy and aging. Cell.

[6] Lapierre LR (2013). The TFEB orthologue HLH-30 regulates autophagy and modulates longevity in *Caenorhabditis elegans*. Nat. Commun..

[7] Hansen M (2008). A role for autophagy in the extension of lifespan by dietary restriction in *C. elegans*. PLoS Genet..

[8] Panowski SH, Wolff S, Aguilaniu H, Durieux J, Dillin A (2007). PHA-4/Foxa mediates diet-restriction-induced longevity of *C. elegans*. Nature.

[9] Greer EL, Brunet A (2009). Different dietary restriction regimens extend lifespan by both independent and overlapping genetic pathways in *C. elegans*. Aging Cell.

[10] Feng Y, Yao Z, Klionsky DJ (2015). How to control self-digestion: transcriptional, post-transcriptional, and post-translational regulation of autophagy. Trends Cell Biol..

[11] Settembre C (2011). TFEB links autophagy to lysosomal biogenesis. Science.

[12] Palmieri M (2011). Characterization of the CLEAR network reveals an integrated control of cellular clearance pathways. Hum. Mol. Genet..

[13] Kenyon CJ (2010). The genetics of ageing. Nature.

[14] Zhang J (2009). Mutation in *Caenorhabditis elegans* Krüppel-like factor, *KLF-3* results in fat accumulation and alters fatty acid composition. Exp. Cell Res..

[15] Zhang J (2011). Regulation of fat storage and reproduction by Krüppel-like transcription factor KLF3 and fat-associated genes in *Caenorhabditis elegans*. J. Mol. Biol..

[16] Riz I, Hawley TS, Hawley RG (2015). KLF4-SQSTM1/p62-associated prosurvival autophagy contributes to carfilzomib resistance in multiple myeloma models. Oncotarget.

[17] Liu C (2015). Impaired autophagy in mouse embryonic fibroblasts null for Krüppel-like Factor 4 promotes DNA damage and increases apoptosis upon serum starvation. Mol. Cancer.

[18] Liao X (2015). Kruppel-like factor 4 is critical for transcriptional control of cardiac mitochondrial homeostasis. J. Clin. Invest..

[19] Carrano AC, Dillin A, Hunter T (2014). A Krüppel-like factor downstream of the E3 ligase WWP-1 mediates dietary-restriction-induced longevity in *Caenorhabditis elegans*. Nat. Commun..

[20] Lapierre LR, Hansen M (2013). Lessons from *C. elegans*: signaling pathways for longevity. Trends Endocrinol. Metab..

[21] Atkins GB, Jain MK (2007). Role of Krüppel-like transcription factors in endothelial biology. Circ. Res..

[22] Hamik A (2007). Kruppel-like factor 4 regulates endothelial inflammation. J. Biol. Chem..

[23] Lakatta EG, Levy D (2003). Arterial and cardiac aging: major shareholders in cardiovascular disease enterprises: part I: aging arteries: a “set up” for vascular disease. Circulation.

[24] Krishnamurthy J (2004). Ink4a/Arf expression is a biomarker of aging. J. Clin. Invest..

[25] Nichols WW (1985). Effects of age on ventricular/vascular coupling. Am. J. Cardiol..

[26] Weiss EP, Fontana L (2011). Caloric restriction: powerful protection for the aging heart and vasculature. Am. J. Physiol. Heart Circ. Physiol..

[27] Torisu T (2013). Autophagy regulates endothelial cell processing, maturation and secretion of von Willebrand factor. Nat. Med..

[28] Torisu K (2016). Intact endothelial autophagy is required to maintain vascular lipid homeostasis. Aging Cell.

[29] Lapierre LR, Kumsta C, Sandri M, Ballabio A, Hansen M (2015). Transcriptional and epigenetic regulation of autophagy in aging. Autophagy.

[30] Kapahi P, Kaberlein M, Hansen M (2016). Dietary restriction and lifespan: lessons from invertebrate models. Ageing Res. Rev..

[31] Hashmi S (2008). A Krüppel-like factor in *Caenorhabditis elegans* with essential roles in fat regulation, cell death, and phagocytosis. DNA Cell Biol..

[32] Rabinowitz JD, White E (2010). Autophagy and metabolism. Science.

[33] Hansen M, Flatt T, Aguilaniu H (2013). Reproduction, fat metabolism, and life span: what is the connection?. Cell Metab..

[34] McConnell BB, Yang VW (2010). Mammalian Krüppel-like factors in health and diseases. Physiol. Rev..

[35] Wang Y (2012). Krüppel-like factor 4 is induced by rapamycin and mediates the anti-proliferative effect of rapamycin in rat carotid arteries after balloon injury. Br. J. Pharmacol..

[36] Zhou G (2012). Endothelial Kruppel-like factor 4 protects against atherothrombosis in mice. J. Clin. Invest..

[37] Liao X (2010). Kruppel-like factor 4 regulates pressure-induced cardiac hypertrophy. J. Mol. Cell. Cardiol..

[38] Dekker RJ (2002). Prolonged fluid shear stress induces a distinct set of endothelial cell genes, most specifically lung Kruppel-like factor 2. Blood.

[39] Parmar KM (2006). Integration of flow-dependent endothelial phenotypes by Kruppel-like factor 2. J. Clin. Invest..

[40] Peng N (2014). An activator of mTOR inhibits oxLDL-induced autophagy and apoptosis in vascular endothelial cells and restricts atherosclerosis in apolipoprotein E−/− mice. Sci. Rep..

[41] Zhang YL (2010). The autophagy-lysosome pathway: a novel mechanism involved in the processing of oxidized LDL in human vascular endothelial cells. Biochem. Biophys. Res. Commun..

[42] Mattart L (2012). The peroxynitrite donor 3-morpholinosydnonimine activates Nrf2 and the UPR leading to a cytoprotective response in endothelial cells. Cell Signal..

[43] Chen G (2013). Hypoxia-induced autophagy in endothelial cells: a double-edged sword in the progression of infantile haemangioma?. Cardiovasc. Res..

[44] Xie Y (2011). Protective role of autophagy in AGE-induced early injury of human vascular endothelial cells. Mol. Med. Rep..

[45] Mueller MA, Beutner F, Teupser D, Ceglarek U, Thiery J (2008). Prevention of atherosclerosis by the mTOR inhibitor everolimus in LDLR-/- mice despite severe hypercholesterolemia. Atherosclerosis.

[46] LaRocca TJ (2012). Translational evidence that impaired autophagy contributes to arterial ageing. J. Physiol..

[47] Chinsomboon J (2009). The transcriptional coactivator PGC-1alpha mediates exercise-induced angiogenesis in skeletal muscle. Proc. Natl Acad. Sci. USA.

[48] Arany Z (2008). HIF-independent regulation of VEGF and angiogenesis by the transcriptional coactivator PGC-1alpha. Nature.

[49] Stiernagle, T. Maintenance of *C. elegans.* WormBook ed. The *C. elegans.* Research Community, WormBook, http://www.wormbook.org (2006). 10.1895/wormbook.1.101.1 PMC4781397 18050451

[50] Mello CC, Kramer JM, Stinchcomb D, Ambros V (1991). Efficient gene transfer in *C. elegans*: extrachromosomal maintenance and integration of transforming sequences. EMBO J..

[51] Greer EL (2007). An AMPK-FOXO pathway mediates longevity induced by a novel method of dietary restriction in *C. elegans*. Curr. Biol..

[52] Robida-Stubbs S (2012). TOR signaling and rapamycin influence longevity by regulating SKN-1/Nrf and DAF-16/FoxO. Cell Metab..

[53] Hsu AL, Murphy CT, Kenyon C (2003). Regulation of aging and age-related disease by DAF-16 and heat-shock factor. Science.

[54] Kenyon C, Chang J, Gensch E, Rudner A, Tabtiang R (1993). A *C. elegans* mutant that lives twice as long as wild type. Nature.

[55] Gerstbrein B, Stamatas G, Kollias N, Driscoll M (2005). In vivo spectrofluorimetry reveals endogenous biomarkers that report healthspan and dietary restriction in *Caenorhabditis elegans*. Aging Cell..

[56] Hughes SE, Evason K, Xiong C, Kornfeld K (2007). Genetic and pharmacological factors that influence reproductive aging in nematodes. PLoS Genet..

[57] Fujioka H, Tandler B, Hoppel CL (2012). Mitochondrial division in rat cardiomyocytes: an electron microscope study. Anat. Rec..

[58] Zhang H (2015). Guidelines for monitoring autophagy in *Caenorhabditis elegans*. Autophagy.

[59] Melendez A (2003). Autophagy genes are essential for dauer development and life-span extension in *C. elegans*. Science.

[60] Feng Z (2006). A *C. elegans* model of nicotine-dependent behavior: regulation by TRP-family channels. Cell.

[61] Li W, Feng Z, Sternberg PW, Xu XS (2006). A *C. elegans* stretch receptor neuron revealed by a mechanosensitive TRP channel homologue. Nature.

[62] Lim YC (2003). Heterogeneity of endothelial cells from different organ sites in T-cell subset recruitment. Am. J. Pathol..

[63] Okuda M (1999). Shear stress stimulation of p130(cas) tyrosine phosphorylation requires calcium-dependent c-Src activation. J. Biol. Chem..

[64] Kirwan JP (1985). Regular exercise enhances insulin activation of IRS-1-associated PI3-kinase in human skeletal muscle. J. Appl. Physiol..

