# Supplementary material for: A conserved KLF-autophagy pathway modulates nematode lifespan and mammalian age-associated vascular dysfunction
Source: Nat Commun. 2017 Oct 13;8:914. doi: 10.1038/s41467-017-00899-5 (PMC5640649; doi:10.1038/s41467-017-00899-5)
Supplement: Supplementary file 1 — Supplementary Information [file 41467_2017_899_MOESM1_ESM.pdf]

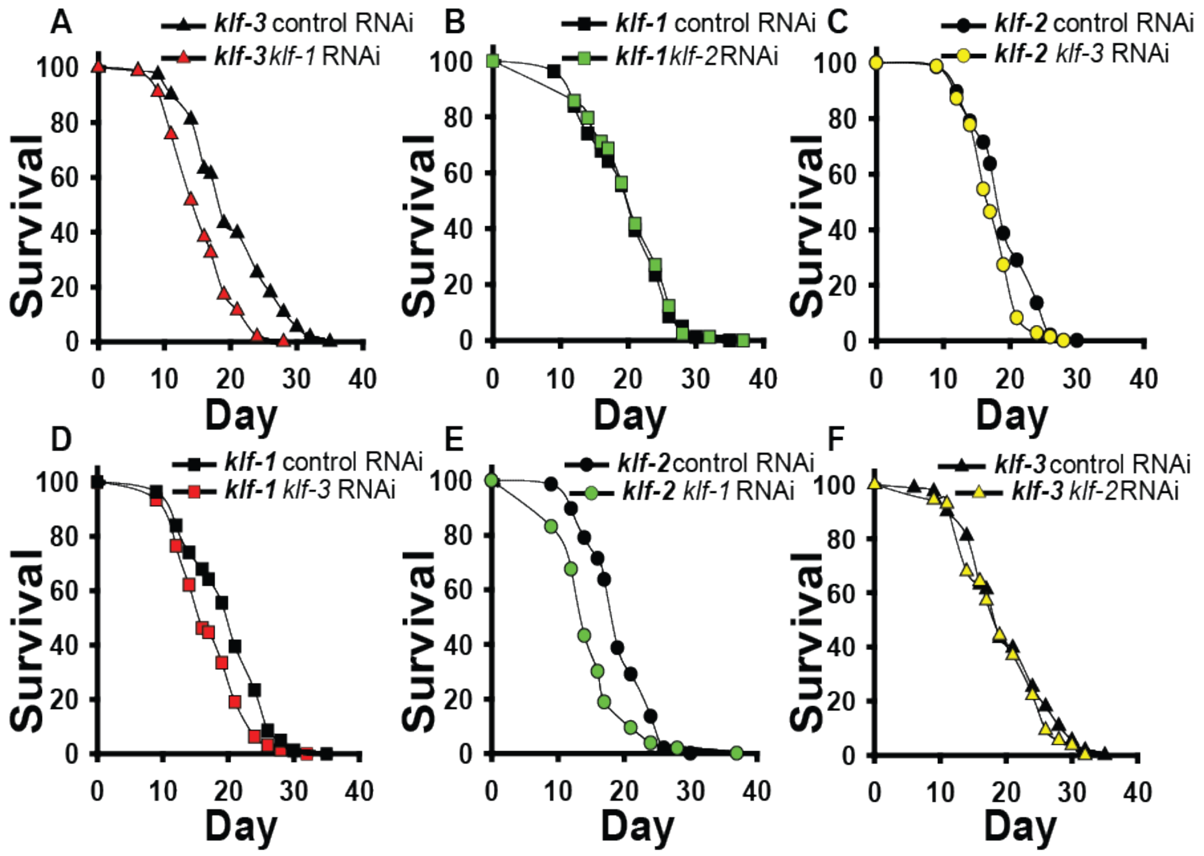

Supplementary Figure 1. Dual loss of function of the Kruppel-like factors reduces *C. elegans* lifespan. (A-F) Lifespan analysis of single KLF mutant worms fed RNAi bacteria targeting a second KLF gene or RNAi bacteria containing empy vector. Bolded genes represent the mutant strain. *Klf-3* mutants are represented with a triangle, *klf-2* with a circle, and *klf-1* with a square. P-value<0.05 by Mantel-Cox log-rank tests compared to control RNAi bacteria. See also Supplementary Tables 1 and 2 for details of lifespan analyses and replicate experiments

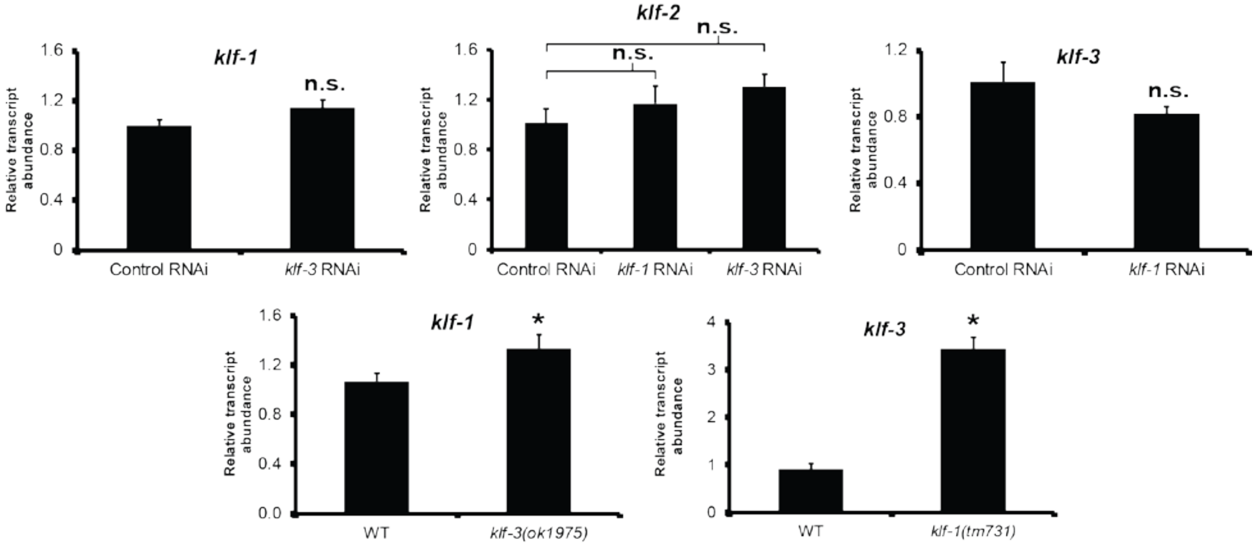

Supplementary Figure 2. Single RNAi depletion of either *klf-1* or *klf-3* is specific and does not alter expression of another *klf*, while mutant worms demonstrate compensatory induction of *klf*s. *klf-1* and *klf-2* transcript levels are nonsignificantly changed by RNAi bacteria feeding targeting *klf-3*, and *klf-2* and *klf-3* transcript levels are nonsignificantly changed by RNAi bacteria feeding targeting *klf-1*. *Klf-1* transcript levels are higher in *klf-3(ok1075)* mutants while *klf-3* is strongly induced in *klf-1(tm731)* mutants. N=3 biological replicates. * represents P-value<0.05, n.s. represents not significant (P-value <0.1) by Students T-test. Error bars represent standard error of the mean.

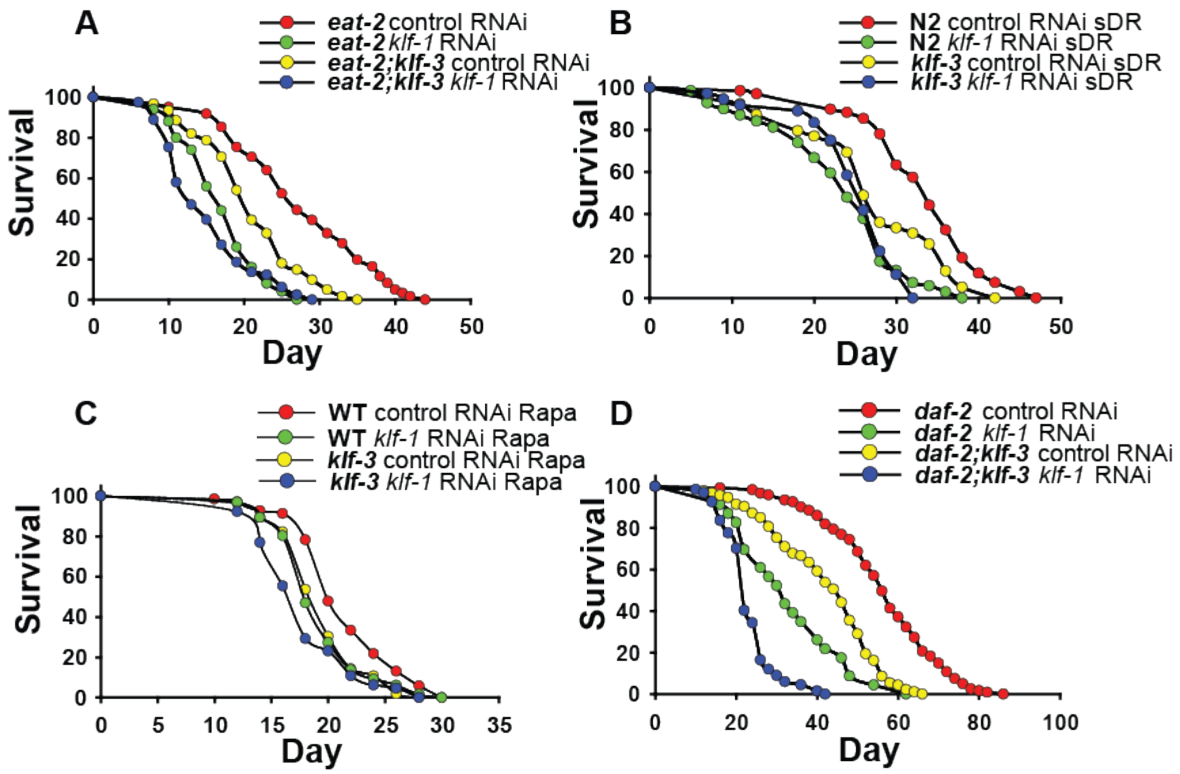

Supplementary Figure 3. Double loss of function of *klf-1* and *klf-3* suppresses enhanced longevity in multiple longevity paradigms. Representative traces are shown here, with full lifespan data and replicates in Supplementary Table 3. Lifespan extension of *eat-2(ad1116)* animals (A), animals subjected to solid agar dietary restriction at 10^8 cfu/mL (sDR) (B), 100uM rapamycin (C), and *daf-2(e1370)* animals (D) is strongly suppressed after double loss of function of *klf-1* and *klf-3*. *klf-3(ok1975)* mutants have mildly suppressed lifespan in *eat-2(ad1116)*, sDR, *daf-2(e1370)* and rapamycin treated animals. RNAi against *klf-1* in rapamycin treated animals had the same lifespan as *klf-3* mutants, but more strongly suppressed lifespan in *eat-2(ad1116)*, sDR, and *daf-2(e1370)* animals. In *eat-2(ad1116)* and sDR animals, RNAi against *klf-1* had similar effects as RNAi against *klf-1* in a *klf-3* mutant background, while in rapamycin treated and *daf-2(e1370)* animals, double loss of function of *klf-1* and *klf-3*

had the strongest suppressive effect on lifespan. See also Supplementary Table 3 for full details on numbers and replicates. Strain is represented in bold. P-value<0.05 by Mantel-Cox log-rank tests.

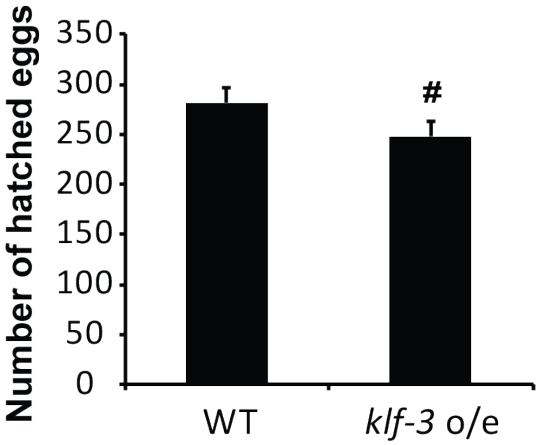

Supplementary Figure 4. Overexpression of *klf-3* does not strongly change total number of hatched eggs laid. Average number of viable eggs laid over the entire life of the N2 animals (281.5±14.2, N=15) compared to *klf-3 o/e* animals (248.0±15.2, N=15). Data is also represented in Fig 2F. # represents P-value<0.1 by Students T-test. Error bars represent standard error of the mean.

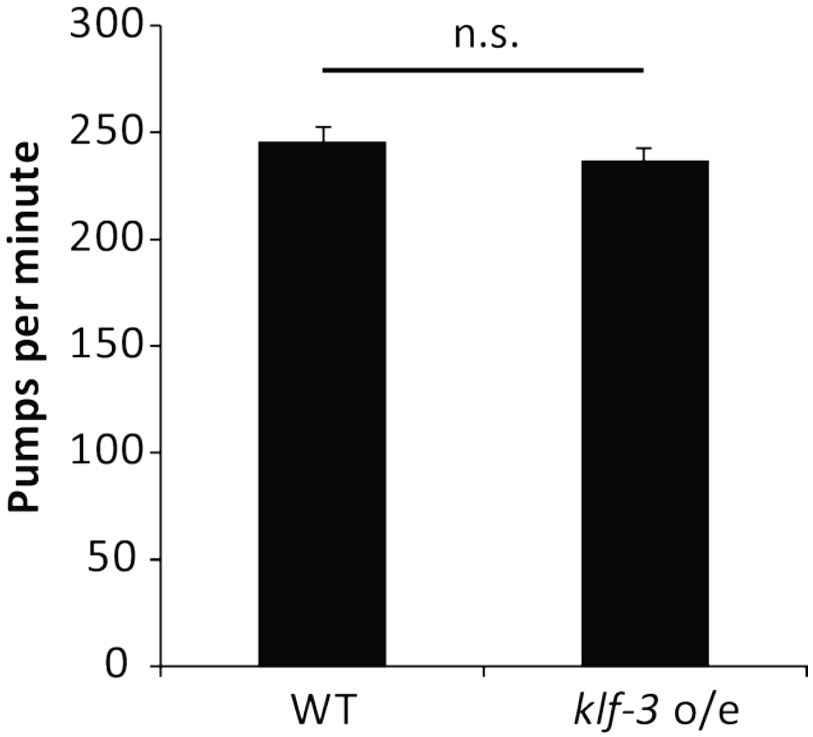

Supplementary Figure 5. Overexpression of *klf-3* does not significantly alter pharyngeal pumping rates. Pharyngeal pumping was assayed in day 1 WT (N=10) and *klf-3 o/e* (N=10) worms on OP50. Further details in Methods. N.S. = not significant. P-value>0.05 by Students T-test. Error bars represent standard error of the mean.

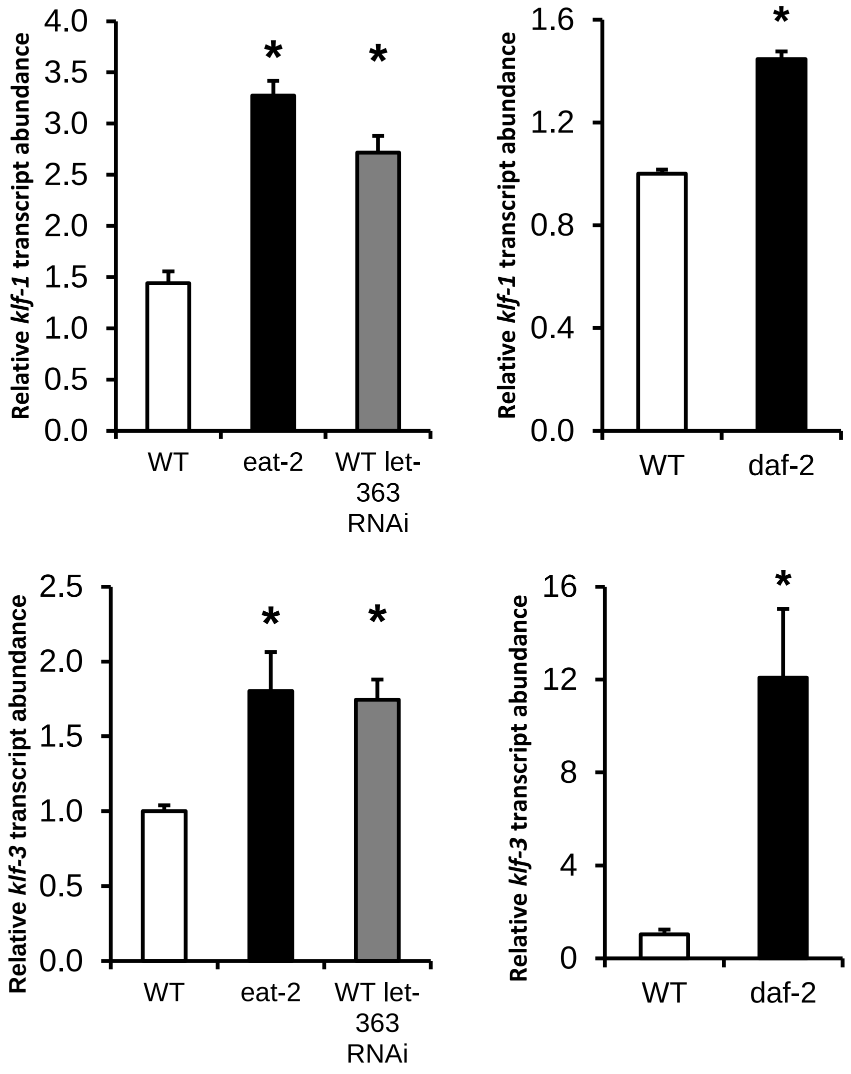

Supplementary Figure 6. Both *klf-1* and *klf-3* are induced in *eat-2* animals and by inhibition of TOR at age Day 12. qPCR analysis of *eat-2(ad1116), daf-2(e1370)* and WT worms subjected to RNAi against *let-363*. Significance determined by Student's t-test, p-value<0.05. N=3 biological replicates. Error bars represent standard error of the mean.

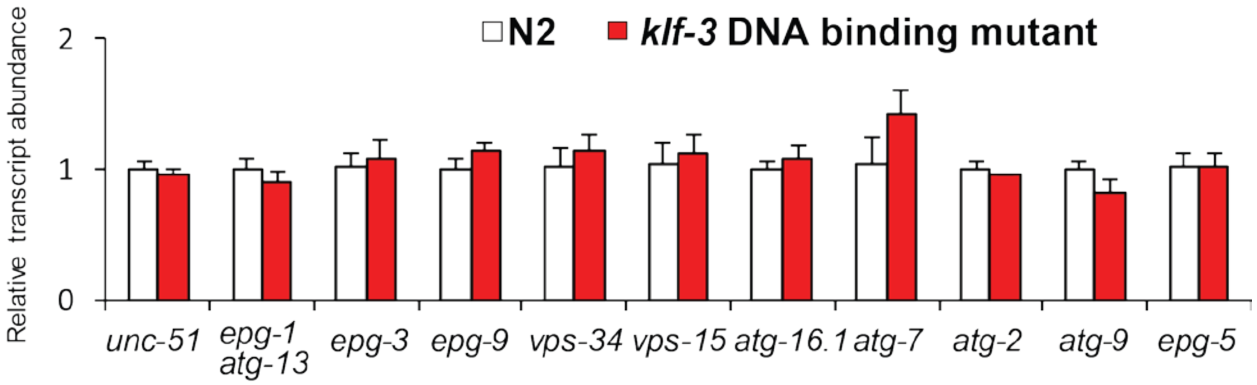

Supplementary Figure 7. Loss of DNA binding region abolishes *klf-3* mediated enhancement of autophagy gene expression. Gene expression of assessed autophagy genes is nonsignificantly different comparing wild-type worms with worms overexpressing *klf-3* with zinc-finger containing region deleted. Significance determined by Student’s t-test, p-value>0.05. N=3 biological replicates. Error bars represent standard error of the mean.

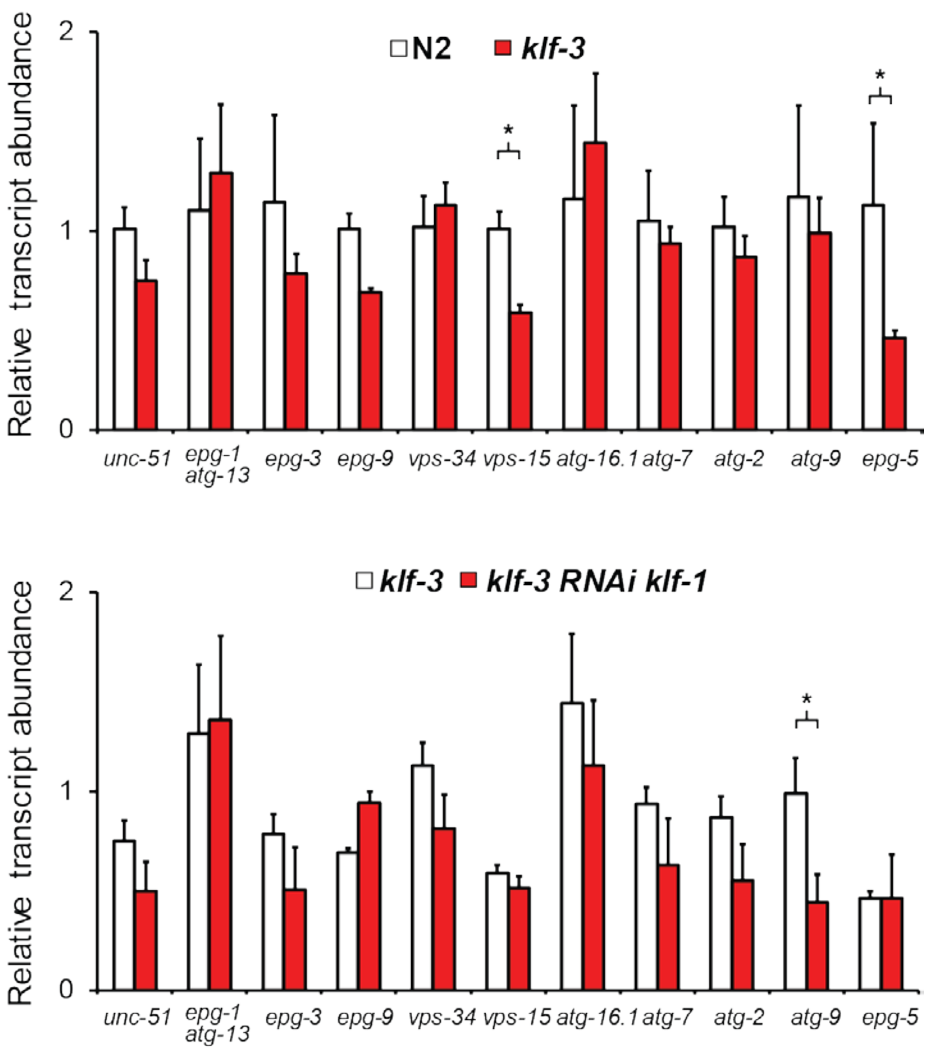

Supplementary Figure 8. *Klf-3* mutants do not strongly suppress autophagy gene expression, and concurrent RNAi inactivation of *klf-1* in *klf-3* mutants weakly reduces expression. Additional comparisons using data displayed in Fig. 3C are shown. Gene expression of assessed autophagy genes is only modestly different comparing wild-type worms with *klf-3(ok1975)* mutants and *klf-3(ok1975)* mutants with *klf-3(ok1975)* mutants fed RNAi targeting *klf-1*. Significance determined by Student's t-test, p-value<0.05. N=3 biological replicates. Error bars represent standard error of the mean.

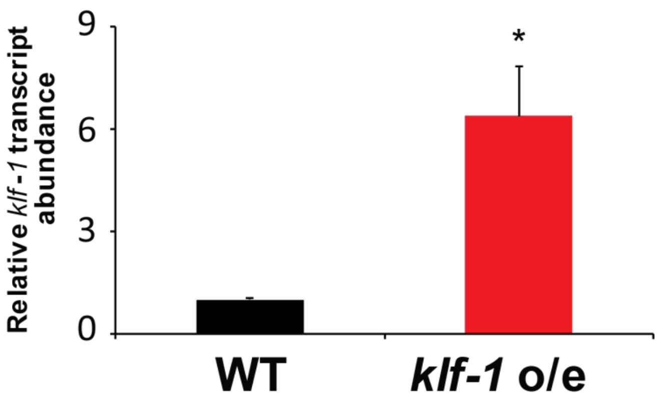

Supplementary Figure 9. *Klf-1* overexpression driven by *ges-1* promoter. *klf-1* transcript levels in *klf-1* o/e animals overexpressing *klf-1* driven by *ges-1* promoter as determined by qPCR (N=3). Significance determined by Student's T-test, p-value <0.05. N=3 biological replicates. Error bars represent standard error of the mean.

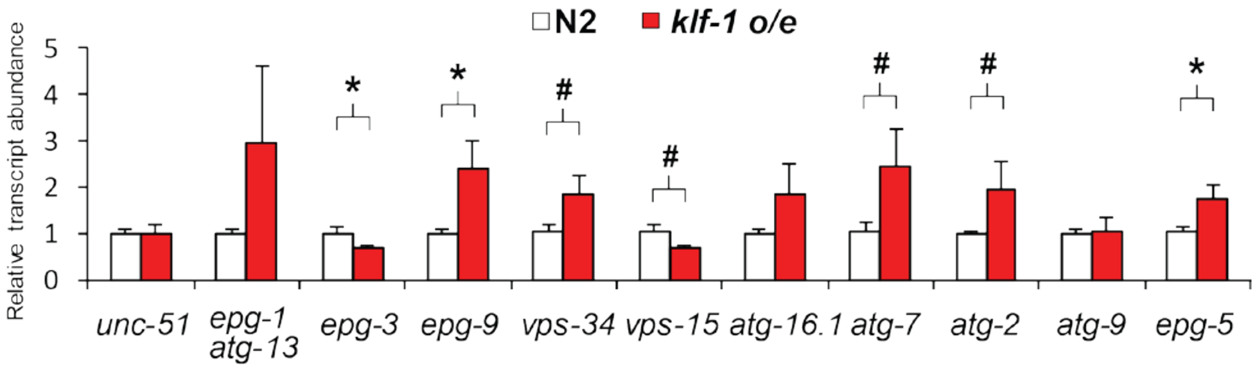

Supplementary Figure 10. *Klf-1* overexpression driven by *ges-1* promoter increases autophagy gene expression. Gene expression of assessed autophagy genes is shown comparing wild-type worms with *klf-1 o/e* worms. Significance determined by Student’s t-test, #p-value<0.01, *p-value<0.05. N=3 biological replicates. Error bars represent standard error of the mean.

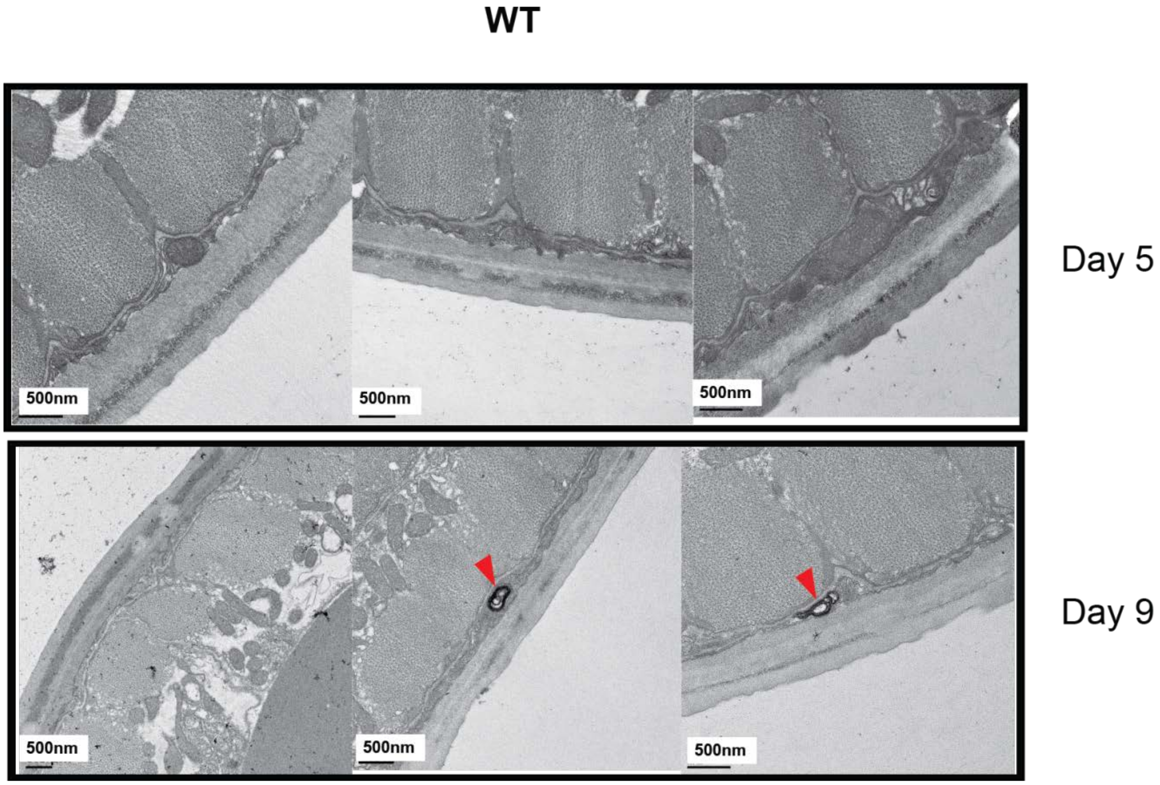
WT
Day 5
Day 9
500nm
500nm
500nm
500nm
500nm
500nm

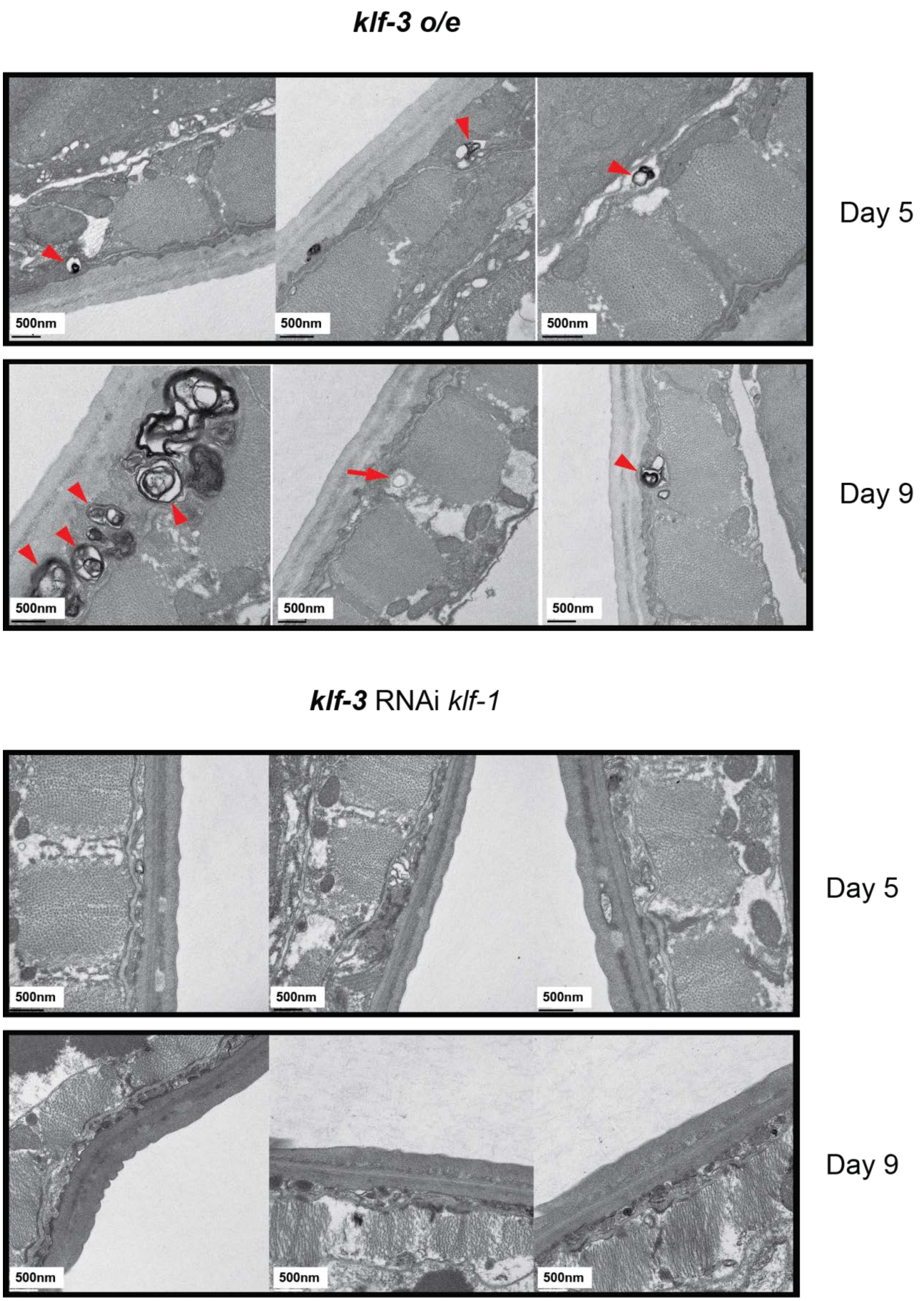

Supplementary Figure 11. Little to no autophagic-like vesicles presence in wild-type and *klf-3* RNAi *klf-1* animals by TEM at Day 5 or Day 9 (post-fertile period).

10-20 transverse sections were taken from animals (n=5) and analyzed; majority of images were void of autophagic vesicles. Sections of *klf-3 o/e* worms are shown for comparison, and are reproduced in Fig. 3F-G. Scale indicated by horizontal bar (500nm), three representative sections from separate animals are shown. Typical autophagic-like vesicle with a double limiting membrane (arrows); autophagic-like vesicle and several autolysosomes (arrowheads).

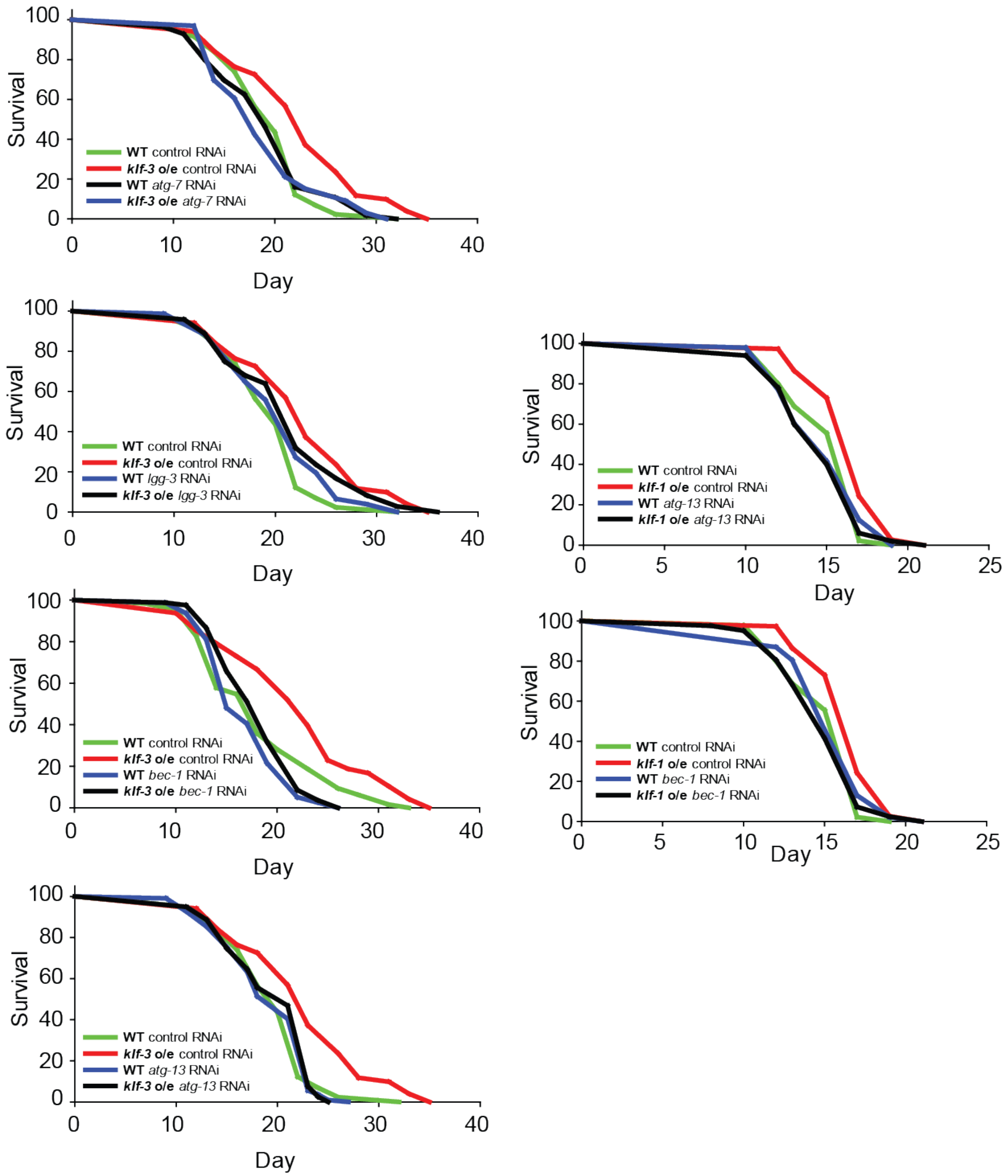

Supplementary Figure 12. KLF-mediated lifespan extension is autophagy dependent. Lifespan analysis of *klf-3 o/e* and *klf-1 o/e* worms fed RNAi targeting empty vector, *bec-1, lgg-3, atg-13*, or *atg-7*. Representative traces are displayed from a single experiment. *Klf-1 o/e* experiments were performed in two independent lines. P-value calculated by Mantel-Cox log-rank tests. Lifespan traces for *klf-3 o/e* and WT with RNAi

against *bec-1* are reproduced from Fig. 3H. See also Supplementary Table 6 for replicates.

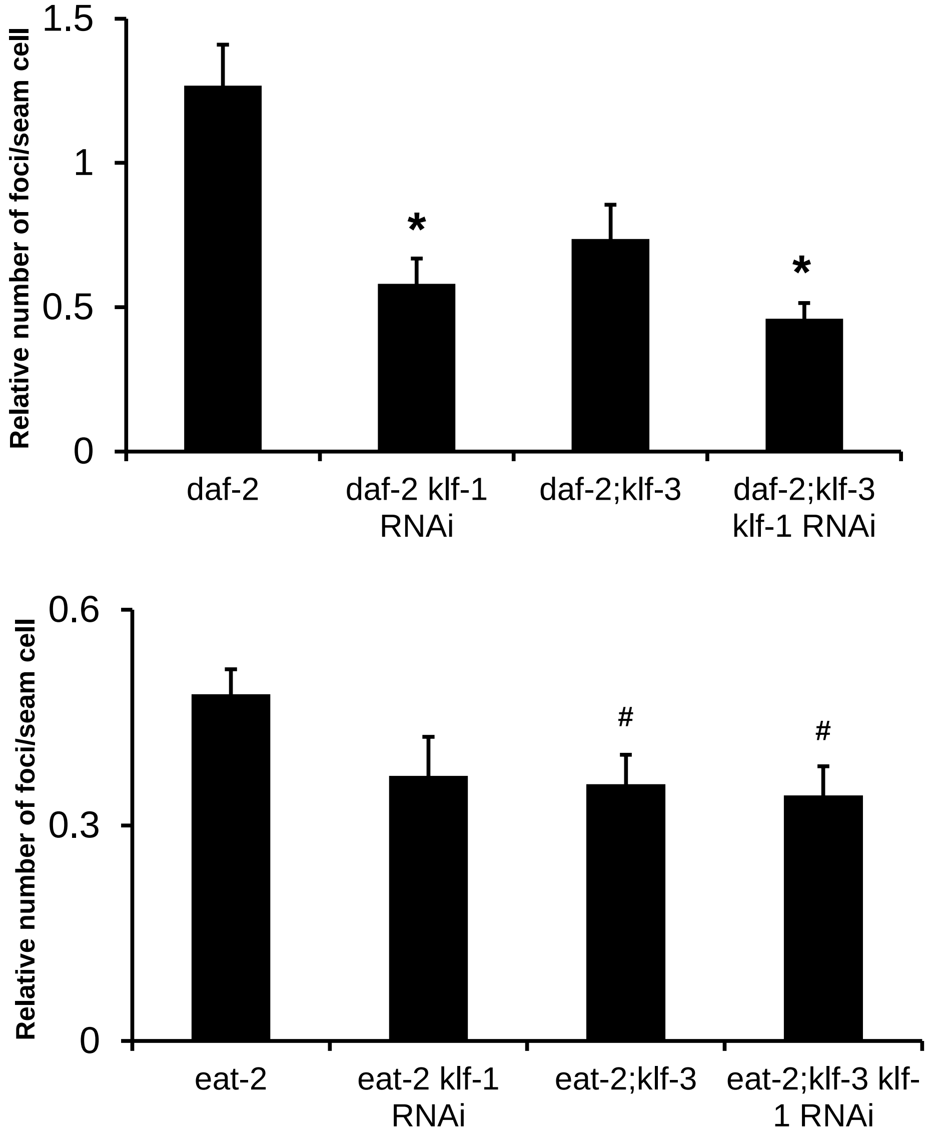

Supplementary Figure 13. Autophagy is decreased in *daf-2* or *eat-2* animals with single or compound deficiency of *klf-1* and *klf-3*. *Daf-2(e1370), eat-2(ad1116), daf-2;klf-3, eat-2;klf-3 animals* were fed *klf-1* RNAi from birth until L4 stage and all images were taken at L4. Compound deficiency of *klf-1* and *klf-3* most severely suppresses autophagy in *daf-2(e1370)* animals, but is nonsignificantly different from single deficiency in *eat-2(ad1116)* animals. 30-150 seams cells were counted in 10-20 animals in each group. * represents P-value<0.05, # represents P-value<0.1 by Students T-test. Error bars represent standard error of the mean.

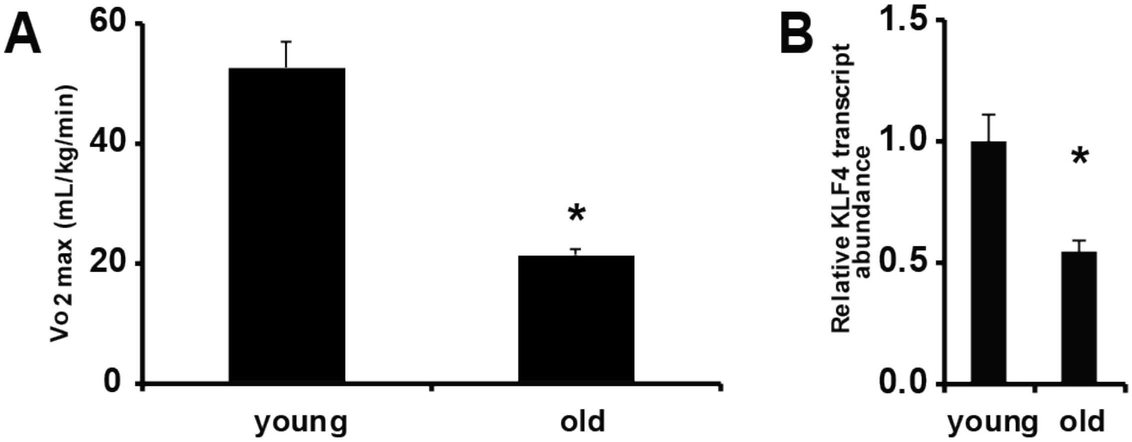

Supplementary Figure 14. VO2max and KLF4 levels decrease with age in humans. (A) Maximal oxygen consumption (VO_{2max}) in young and old patients. N=5 patients per group. * represents P-value<0.05 by Students T-test. Further details in Methods Section. (B) qPCR analysis of skeletal muscle biopsies (whole tissue) in young (20-40 years old) and aged (50-70 years old) patients. N=5 patients per group. * represents P-value<0.05 by Students T-test. Error bars represent standard error of the mean.

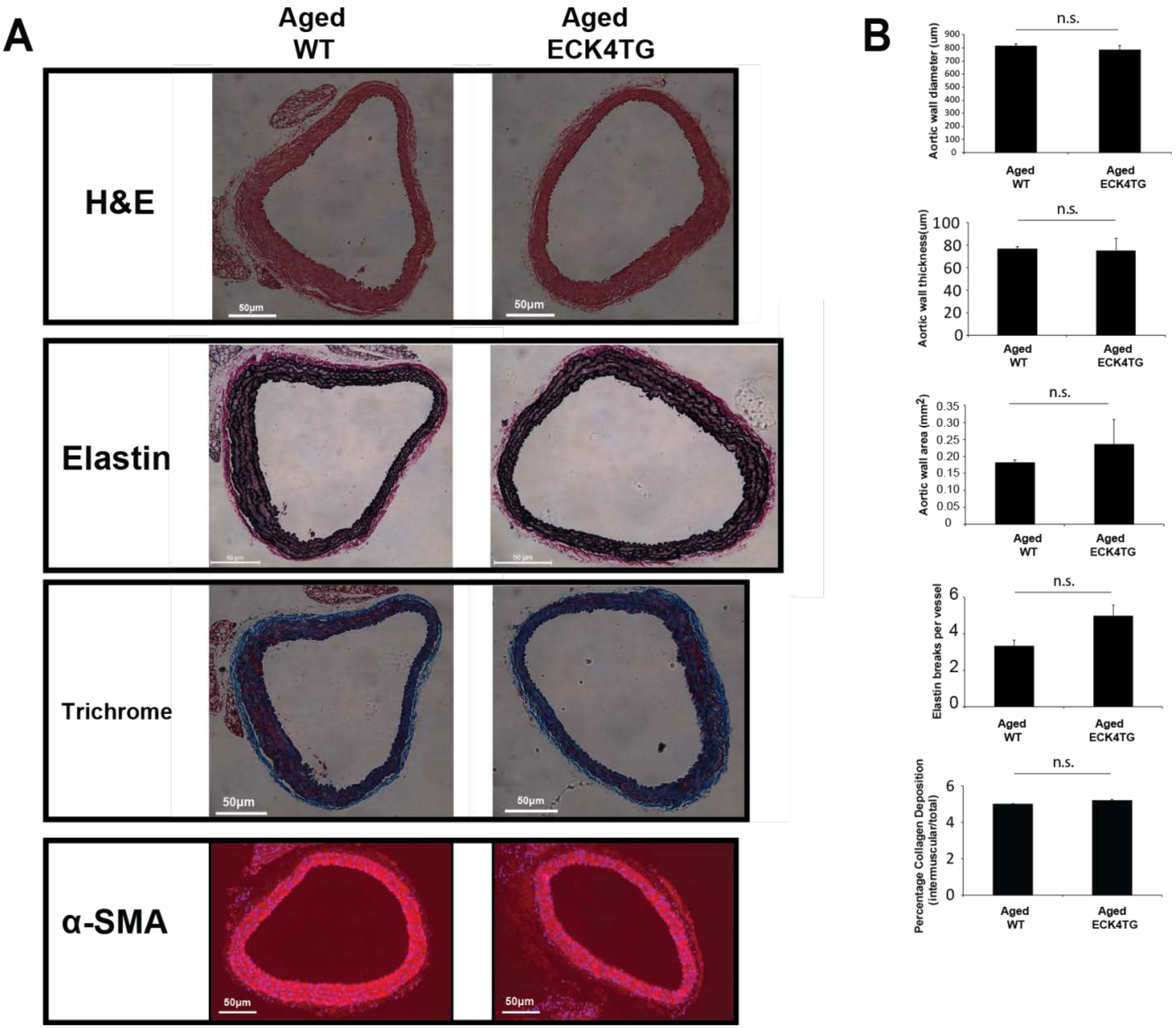

Supplementary Figure 15. Structural wall components are unchanged in aged WT versus aged ECK4TG mice.
(A) Representative aortic sections from aged (12-14 months) WT and ECK4TG mice, N=3 per group. Top, hematoxylin and eosin staining, upper middle, elastin staining, lower middle, trichrome staining, bottom, antibody against α-smooth muscle actin (red) with 4′,6-diamidino-2-phenylindole (DAPI, blue) counterstaining to identify nuclei. Sections were taken at the T2 level of the thoracic aorta. All images were taken at 100x magnification. Scale bar, 50μm. (B) Quantitative morphometry (lumen diameter, aortic wall thickness, aortic wall area), and quantification of elastin degradation and collagen deposition of perfusion-fixed aortic cross sections from aged (12-14 months) WT and ECK4TG mice, N=3 per group. N.S. = non-significant by Student’s T-test. Further details in Methods Section. Error bars represent standard error of the mean.

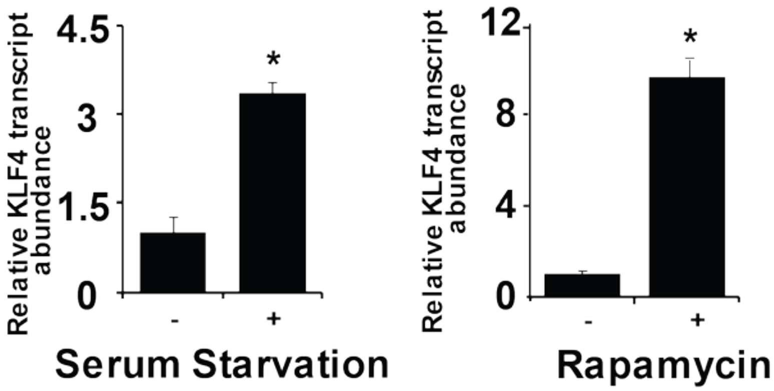

Supplementary Figure 16. KLF4 is induced in HUVECs by serum starvation and rapamycin. Rapamycin treatment (20ug/mL) (A) and serum starvation (B) induce KLF4 expression. Cells were incubated in rapamycin 24 hours before harvest. Starvation was accomplished via replacement of media with DPBS for the 1 hour before harvest. *P-value<0.05 by Student's T-test. N=3 biological replicates. Error bars represent standard error of the mean.

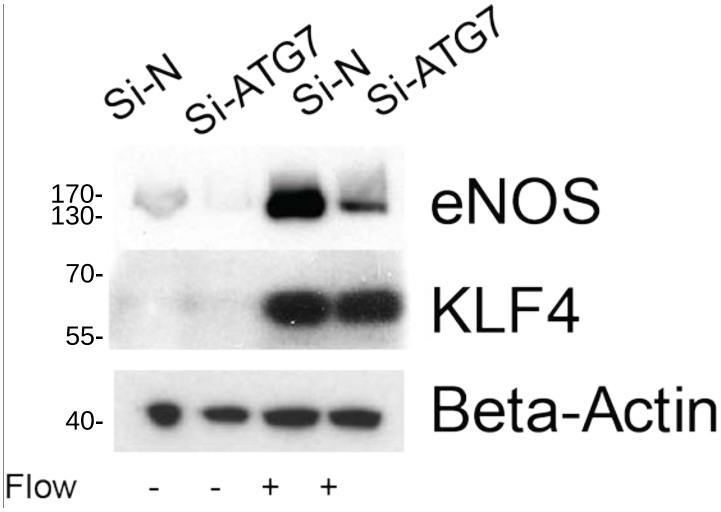

Supplementary Figure 17. Flow-induced eNOS expression is autophagy dependent HUVECs were subjected to siRNA against *Atg7* for 48 hours, then to static or laminar flow (shear stress of 17 dyne/cm^2) for an additional 24 hours prior to harvest. Representative blot is shown from two independent experiments. Further details in Methods section.

A

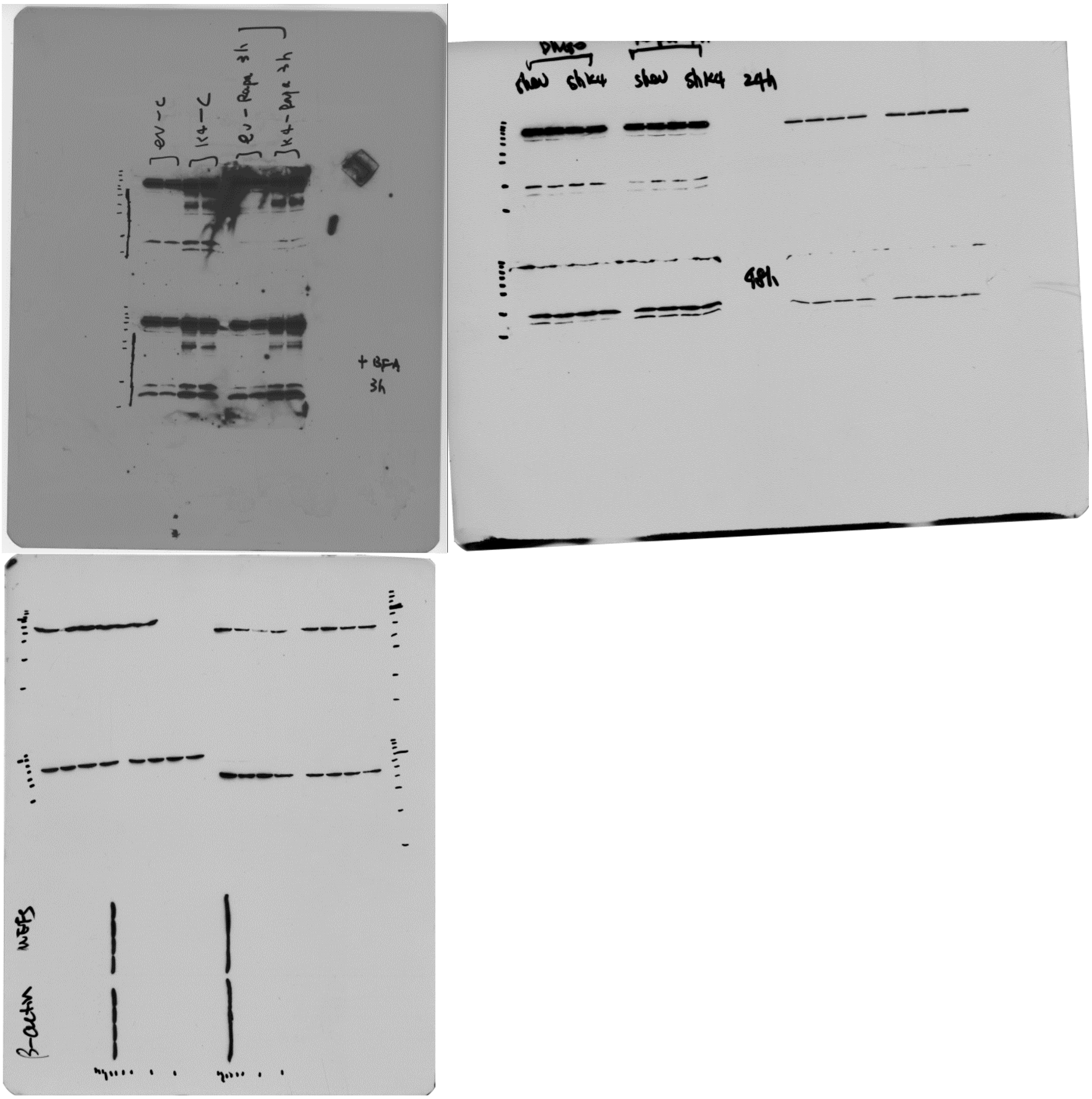

B

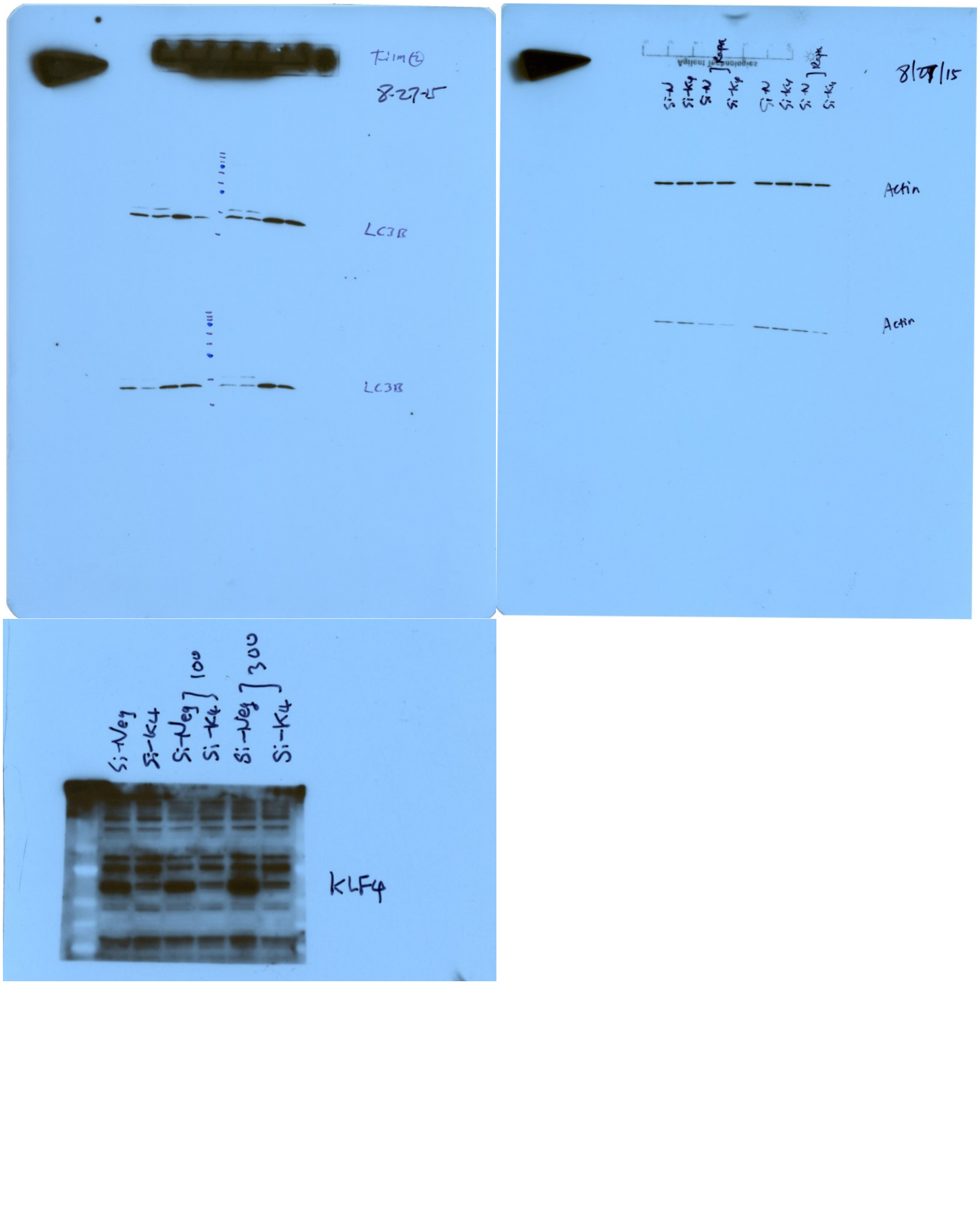
8-27-15
LC3B
LC3B
Actin
Actin
Si-Neg
Si-K4
Si-Neg
Si-K4
100
Si-Neg
300
Si-K4
KLF4

C

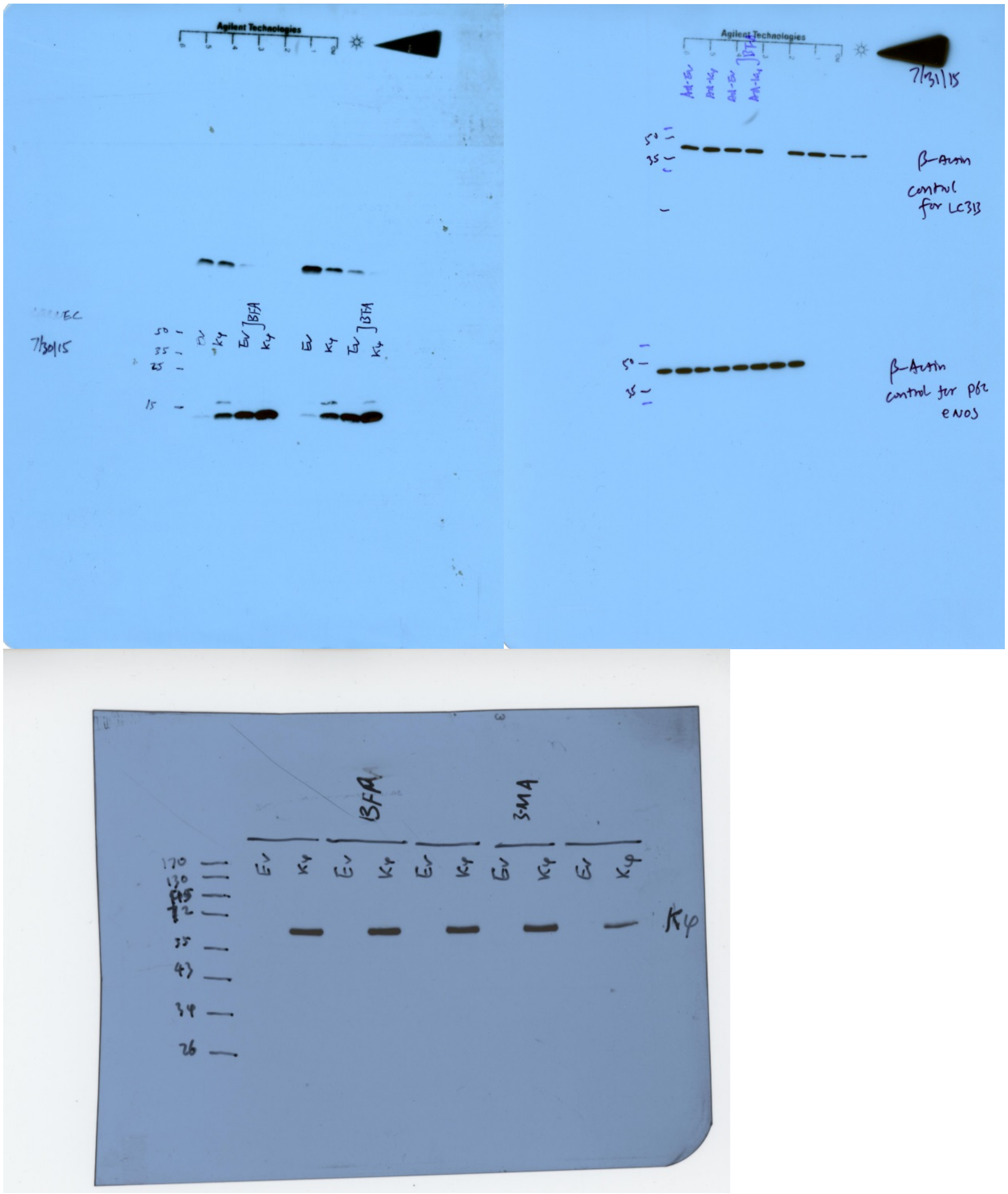
Agilent Technologies
7/30/15
BFA
Agilent Technologies
7/31/15
β-Actin
control
for LC3B
β-Actin
control for p62
eNOS
BFA
3-MA
170
130
35
43
34
26

D

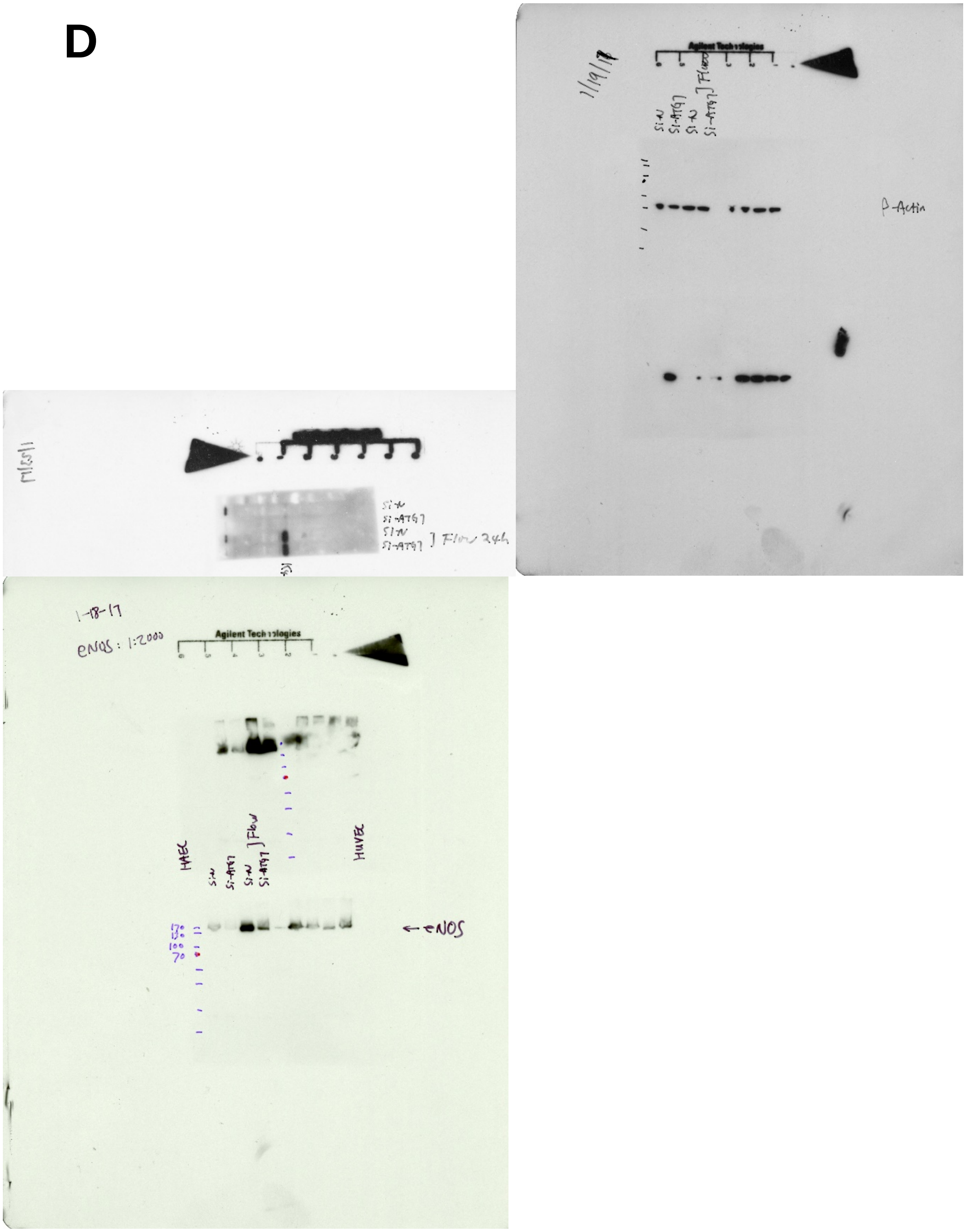

Supplementary Figure 18. Full scans of western blots. (A) LC3-I lipidation assay in MEFs with overexpression of knockdown of KLF4. Cropped data presented in Figure 4C (B) LC3-I lipidation assay in HUVECs with knockdown of KLF4. Cropped data presented

in Figure 5G (C) LC3-I lipidation assay in HUVECs with overexpression of KLF4. Cropped data presented in Figure 5G. (D) Western blots of eNOS, KLF4 and actin expression with and without laminar shear stress. Cropped data presented in Supplementary Figure 17.

RNAi started in adulthood	Vector Control RNAi	*klf-1(* RNAi	*klf-2* RNAi	*klf-3* RNAi
**N2**		↓	—	—
klf-1(tm731)	—	NA	—	↓↓
klf-2(ok1043)	—	↓↓	NA	↓
klf-3(ok1975)	—	↓↓	—	NA

RNAi started from hatching	Vector Control RNAi	*klf-1* RNAi	*klf-2* RNAi	*klf-3* RNAi
**N2**		↓	—	Lethal
klf-1(tm731)	—	NA	↓	Lethal
klf-2(ok1043)	—	↓↓	NA	Lethal
klf-3(ok1975)	—	↓↓↓	—	NA

Supplementary Table 1. Summary table of combinatorial lifespan analysis of double KLF loss of function. Complete data in Supplementary table 2. Each single arrow represents approximately 10% loss of lifespan. Horizontal bars represent nonsignificant difference. NA = not attempted.

Strains	RNAi treatments	Avg lifespan ± s.e.m. (Days)	Number of animals	25% (Days)	50% (Days)	75% (Days)	% change	P-value vs control
N2	OP50	18.586 ± 0.781	65/90	23	18	14		
	Vector control	18.608 ± 0.661	79/86	23	18	14		
	klf-1	16.322 ± 0.630	68/90	20	14	12	-12	**0.017**
		15.988 ± 0.590	81/90	18	14	12	-14	**0.004**
	klf-2	17.934 ± 0.588	68/90	23	16	14	-4	0.388
		19.638 ± 0.629	77/90	23	20	16	+6	0.443
	klf-3	17.927 ± 0.819	48/90	23	16	14	-4	0.540
klf-1 (tm731)	OP50	19.869 ± 0.608	81/90	24	21	14	+7	0.528
	Vector control	19.869 ± 0.608	81/90	24	21	14		
	klf-2	20.432 ± 0.571	82/90	26	21	16	+3	0.490
	klf-3 (from birth)	lethal						
	(from adult)	17.336 ± 0.608	65/90	21	16	14	-13	**0.002**
klf-2(ok1043)	OP50	19.250 ± 0.586	56/90	24	19	16	+4	0.893
	Vector control	19.236 ± 0.588	56/90	24	19	16		
		19.161 ± 0.470	57/90	22	22	19		
	klf-1 (from birth)	15.661 ± 0.585	40/90	19	16	13	-18	**<0.001**
	(from adult)	15.149 ± 0.617	56/90	17	14	12	-20	**<0.001**
	klf-3 (from birth)	Lethal						
	(from adult)	17.593 ± 0.424	74/90	21	17	16	-9	**0.012**
klf-3(ok1976)	OP50	19.829 ± 0.714	57/90	26	19	16	+7	0.478
	Vector control	19.829 ± 0.714	57/90	26	19	16		
		18.466 ± 0.642	55/90	22	22	16		
	klf-1(from birth)	13.681 ± 0.567	38/90	16	13	10	-26	**<0.001**
	(from adult)	15.876 ± 0.618	55/90	19	16	14	-20	**<0.001**
	klf-2 (from birth)	19.000 ± 0.364	60/90	22	19	19	+3	0.362
	(from adult)	19.344 ±	56/90	24	19	14	-2	0.420

	0.715

Supplementary Table 2. Combinatorial lifespan analysis of *C. elegans* animals with reduced *klf-1*, *klf-2* or *klf-3* levels. Results of lifespan analysis of wild-type (WT, N2) and mutant animals (*klf-1, klf-2* and *klf-3*). Animals were raised and incubated at 20ºC and fed control bacteria or bacteria expressing dsRNA against *klf-1, klf-2* or *klf-3* beginning at adulthood. The *klf-1(tm731)* mutant contains a 343-bp intronic deletion with no obvious abnormalities, likely hypomorphic. The *klf-2(ok1043)* mutant contains an estimated 1500-bp deletion. The *klf-3(ok1975)* loss-of-function mutant contains a 1658-bp deletion spanning the 3' end of exon 2 through the 5' end of exon 3. Data show the average lifespan, number of events, day at which 25, 50 or 75% of animals remained alive, % change, and p value vs control calculated by Mantel-Cox log-rank test.

Strains/Regimen	RNAi treatments	Avg lifespan ± s.e.m. (Days)	Number of animals	25% (Days)	50% (Days)	75% (Days)	% change	P-value vs control
N2 Rapamycin	Vector Control	21.594±0.517	69/90	24	20	20		
		23.253±0.741	79/90	27	23	17		
		24.407±0.637	81/90	28	26	21		
	klf-1	19.424±0.463	66/90	22	18	18	-10	**0.0070**
		19.355±0.588	62/90	21	19	17	-16	**<0.001**
		18.290±0.533	69/90	21	18	14	-25	**<0.001**
klf-3 Rapamycin	Vector Control	19.571±0.477	56/90	22	20	18	-9	**0.0076**
		20.534±0.680	58/90	23	19	17	-11	**0.0140**
		18.729±0.660	48/90	21	18	16	-23	**<0.001**
	klf-1	17.969±0.493	65/90	20	18	16	-17	**<0.001**
		17.552±0.423	67/90	19	17	17	-25	**<0.001**
		18.268±0.439	71/90	21	18	16	-25	**<0.001**
N2 solid agar dietary restriction (sDR)	Vector Control	27.081±1.383	74/90	34	26	15		
		27.043±0.980	70/90	32	28	22		
		33.368±0.876	68/90	38	34	30		
	klf-1	20.652±1.074	69/90	26	20	13	-24	**0.0020**
		22.020 ± 0.906	50/90	26	22	18	-18	**0.0013**
		23.116 ± 0.961	69/90	28	24	18	-31	**<0.001**
klf-3 sDR	Vector Control	22.197±1.25	66/90	30	20	13	-18	**0.0595**
		26.143±1.318	49/90	34	26	20	-3	0.964
		26.949±1.457	39/90	36	26	22	-19	**0.0022**
	klf-1	21.485±1.030	66/90	28	20	15	-21	**0.0060**
		26.667±1.048	51/90	32	26	22	-1	0.992
		24.861±1.005	36/90	28	26	22	-25	**<0.001**
eat-2	Vector Control	27.508 ±0.985	61/90	34	28	22		
		25.648±1.062	54/90	31	25	19		
		23.702±0.838	57/90	28	24	18		
eat-2	*klf-1*	20.551±0.621	69/90	24	22	18	-19	**<0.001**
		16.920±0.702	50/90	21	17	13	-34	**<0.001**
		16.821±0.710	56/90	18	16	11	-29	**<0.001**
eat-2;klf-3	Vector Control	19.130±0.936	46/90	24	18	14	-30	**<0.001**
		20.770±0.832	61/90	25	21	17	-20	**<0.001**
		19.875±1.175	24/90	22	18	16	-16	**0.0458**
	klf-1	8.435±0.677	23/90	8	8	6	-70	**<0.001**
		14.975±0.648	81/170	19	13	11	-42	**<0.001**
		18.821 ± 1.104	39/90	26	18	13	-20	**0.0085**
daf-2	Vector Control	56.083±1.244	121/150	66	56	48		
		50.101±1.584	79/90	62	52	40		

	klf-1	33.043±2.652	23/150	42	32	22	-41	**<0.001**
		21.5±1.23	36/90	26	20	16	-57	**<0.001**
daf-2;*klf-3*	Vector Control	41.849±1.359	93/150	52	46	32	-25	**<0.001**
		34.364±3.334	11/90	44	38	20	-31	**<0.001**
	klf-1	23.075±0.759	67/150	26	22	20	-59	**<0.001**
		17.143±0.957	35/90	20	16	14	-66	**<0.001**

Supplementary Table 3: Lifespan analysis of *C. elegans* mutant animals with reduced *klf-1* levels. Results of lifespan analysis of wild-type (WT, N2) and *klf-3* mutant animals. Animals were raised and incubated at 20ºC and fed control bacteria or bacteria expressing dsRNA against *klf-1* beginning at adulthood. Data show the average lifespan, number of events, day at which 25, 50 or 75% of animals remained alive, % change, and p value vs control calculated by Mantel-Cox log-rank test.

Strains (ng of plasmid injected)	Avg lifespan ± s.e.m. (Days)	Number of animals	25% (Days)	50% (Days)	75% (Days)	% change	P-value vs control
N2	*17.683 ± 0.381	69/90	20	20	15		
	#17.096 ± 0.423	78/90	20	20	15		
	†17.107 ± 0.368	74/90	20	20	15		
	‡21.079 ± 0.409	64/90	26	23	18		
	Δ14.946 ± 0.505	49/60	17	15	13		
	º19.563 ± 0.395	131/180	22	20	16		
	ǂ19.739 ± 0.636	33/90	22	20	18		
	ϕ21.128 ± 0.413	148/166	24	22	18		
	ε14.568±0.433	44/60	17	15	12		
	ζ14.370±0.399	54/60	17	13	12		
	θ13.159±0.409	44/60	15	13	11		
	κ17.452 ± 0.266	146/171	20	17	15		
	λ10.859 ± 0.313	149/160	13	9	8		
klf-3 o/e 10ng	***Line #1** 16.578 ± 0.372	79/90	20	16	14	-6	0.162
	**#Line #2** 16.345 ± 0.396	64/90	20	16	14	-4	0.268
	**†Line #3** 16.532 ± 0.414	69/90	20	16	14	-3	0.744
klf-3 o/e 50ng	**‡Line #1** 22.673 ± 0.857	51/90	26	23	18	+8	**0.007**
	**ΔLine #1** 16.333 ± 0.473	51/60	19	17	15	+9	**0.050**
	**º Line #2** 21.760 ± 0.661	99/180	26	22	16	+11	**<0.001**
	**ǂLine #2** 26.390 ± 1.774	17/60	31	27	21	+34	**<0.001**
Mutant *klf-3* o/e 50ng	ϕ **Line #1** 20.456 ± 0.320	171/183	24	22	18	-3	0.056
	**εLine #2** 13.956 ± 0.384	45/60	17	13	12	-4	0.250
	**ζLine #2** 13.233 ± 0.556	30/60	15	13	11	-8	0.130
	**θLine #2** 13.479 ± 0.367	48/60	15	12	12	+2	0.633

Supplementary Table 4: Lifespan analysis of *C. elegans* with *klf-3* over-expression (o/e). Results of lifespan analysis of wild-type (WT, N2) and *klf-3* overexpressing animals. Animals were raised and incubated at 20ºC and fed OP50 E. coli. Data show the average lifespan, number of events, day at which 25, 50 or 75% of animals remained alive, % change, and p value vs control calculated by Mantel-Cox log-rank test. Symbols represent experimental and control groups used to calculate p-values.

**Gene**	**Mammalian Ortholog**	**Position from start codon**
unc-51	*ulk1*	-11
epg-1	*atg13*	-400
		-450
epg-3	*vmp1*	-547
		-882
epg-9 isoform b	*atg101*	-586
		-590
vps34 isoform c	*pik3c3*	4
vps15 isoform a	*pik3r4*	151
		-956
vps15 isoform b	*pik3r4*	-356
		-385
atg16.1	*atg16L1, atg16L2*	48
atg-7	*atg7*	-1055
atg-2	*atg2*	56
		-610
		-655
atg-9 isoform a	*atg9*	-108
		-173
atg-9 isoform b		-1017
epg-5	*epg5*	61

Supplementary Table 5: In silico search identifies presence of KLF response elements GA/GCCC within 1000 base pairs upstream and 200 base pairs downstream of start codon in autophagy genes. Promoters of autophagy related genes were manually searched for presence of GACCC or GGCCC boxes using UCSD Genome browser, and positions indicated relative to start codon.

Strains	RNAi	Avg lifespan ± s.e.m.	Number of	25%	50%	75%	%	P-value

	treatments	(Days)	animals	(Days)	(Days)	(Days)	change	vs control
N2	Vector control	*18.329±0.749	64/90	23	18	14		
N2	*bec-1*	*17.038 ± 0.426	79/90	19	15	15	-7	**<0.0001**
klf-3 o/e	Vector control	*21.985±1.001	48/90	25	23	16	+20	**0.029**
klf-3 o/e	*bec-1*	*17.769 ± 0.541	70/90	21	18	14	-19	**<0.0001**
klf-3 o/e	*bec-1*	*18.198 ± 0.420	82/90	22	19	15	-17	**<0.0001**
klf-3 o/e	*bec-1*	*13.711 ± 0.334	76/90	16	14	12	-38	**<0.0001**
N2	Vector control	#18.685±0.597	73/90	22	18	14		
N2	*bec-1*	#15.221±0.359	76/90	18	16	12	-18	**<0.0001**
klf-3 o/e	Vector control	#21.462 ± 0.688	78/90	24	22	18	+15	**0.0024**
N2	Vector control	†20.457±0.649	81/90	24	19	15		
N2	*lgg-3*	†20.469 ± 0.576	77/90	24	22	17	<0.1	0.989
N2	*atg-7*	†20.067 ± 0.681	56/91	22	22	17	-2	0.816
N2	*atg-13*	†19.138±0.407	109/120	23	21	17	-6	**0.00851**
klf-3 o/e	Vector control	†22.802 ± 0.768	66/90	29	22	17	+11	**0.0273**
klf-3 o/e	*lgg-3*	†21.426 ± 0.702	72/90	24	22	17	-6	0.0567
klf-3 o/e	*atg-7*	†21.035 ± 1.013	58/91	29	22	15	-8	0.845
klf-3 o/e	*atg-7*	†19.030 ± 0.876	33/90	21	18	14	-16	**0.0161**
klf-3 o/e	*atg-13*	†19.519±0.464	79/120	23	21	15	-14	**<0.0001**
N2	Vector control	Δ15.441±0.595	59/60	20	14	12		
N2	*lgg-3*	Δ15.628±0.650	43/60	20	16	12	+1	0.956
N2	*atg-7*	Δ16.370±0.609	54/60	20	16	14	+6	0.306
klf-3 o/e	Vector control	Δ17.830±0.611	53/60	20	20	14	+15	**0.011**
klf-3 o/e	*lgg-3*	Δ14.578±0.605	45/60	18	14	12	-18	**<0.001**
klf-3 o/e	*atg-7*	Δ15.765±0.645	51/60	20	14	12	-12	**0.030**

Strains	RNAi treatments	Avg lifespan ± s.e.m. (Days)	Number of animals	25% (Days)	50% (Days)	75% (Days)	% change	P-value vs control
N2	Vector control	*15.289±0.337	45/60	17	17	13		
N2	*bec-1*	***Line #1** 15.696±0.326	46/60	17	15	15	+3	0.579
N2	*bec-1*	***Line #1** 15.896±0.335	48/60	18	16	14	+4	0.081
N2	*atg-13*	***Line #1** 15.021±0.364	48/60	17	15	13	-2	0.778
N2	*atg-13*	***Line #1** 16.735±0.419	49/60	18	16	14	+9	**0.002**
klf-1 o/e	Vector control	***Line #1** 16.703±0.340	37/60	17	17	15	+9	**0.004**
klf-1 o/e	*bec-1*	***Line #1** 15.049±0.407	41/60	17	15	13	-10	**0.005**
klf-1 o/e	*atg-13*	***Line #1** 14.820±0.360	50/60	17	15	13	-11	**0.001**
N2	Vector control	#15.795±0.432	39/60	18	16	14		
N2	*bec-1*	**#Line #2** 15.615±0.415	26/60	18	16	14	-1	0.670
N2	*atg-13*	**#Line #2** 17.250±0.565	36/60	18	18	16	+9	**0.009**
klf-1 o/e	Vector control	**#Line #1** 16.180±0.429	50/60	18	16	14	+6	**0.004**
klf-1 o/e	*bec-1*	**#Line #1** 14.920±0.329	50/60	16	14	14	-8	**0.007**
klf-1 o/e	*atg-13*	**#Line #1** 16.265±0.353	49/60	18	16	14	-1	0.931

N2	Vector control	†16.020±0.367	51/60	18	16	14		
N2	*bec-1*	**†Line #2** 15.821±0.311	56/60	18	16	14	-1	0.490
N2	*atg-13*	**†Line #2** 16.634±0.501	41/60	18	18	14	+4	0.119
klf-1 o/e	Vector control	**†Line #2** 18.105±0.475	38/60	21	18	16	+14	**<0.0001**
klf-1 o/e	*bec-1*	**†Line #2** 14.941±0.494	34/60	18	16	12	-17	**<0.001**
klf-1 o/e	*atg-13*	**†Line #2** 16.389±0.307	36/60	18	16	16	-9	**0.001**
klf-1 o/e	Vector control	**‡Line #2** 18.113±0.332	71/90	21	18	16	+13	**<0.0001**
klf-1 o/e	*bec-1*	**‡Line #2** 15.468±0.285	79/90	18	16	14	-15	**<0.0001**
klf-1 o/e	*atg-13*	**‡Line #2** 16.717±0.384	53/60	18	18	14	-8	**0.024**

Supplementary Table 6: Lifespan analysis of *C. elegans klf-3* and *klf-1 o/e* animals with reduced *beclin-1, lgg-3*, *atg-13* or *atg-7* levels. Results of lifespan analysis of wild-type (WT, N2), *klf-1 o/e* and *klf-3 o/e* animals with reduced *beclin-1, lgg-3, atg-13* or *atg-7* levels. Animals were raised and incubated at 20ºC and fed control bacteria or bacteria expressing dsRNA against *bec-1, lgg-3, atg-13* or *atg-7* beginning at adulthood. Data show the average lifespan, number of events, day at which 25, 50 or 75% of animals remained alive, % change, and p value vs control calculated by Mantel-Cox log-rank test. *,#,†,‡ symbols represent experimental and control groups used to calculate p-values.

		**Ad-KLF4**		**Si-KLF4**	
**Gene Symbol**	**Gene**	**Fold change**	**p-value**	**Fold change**	**p-value**
Akt1	Akt1 Thymoma viral proto-oncogene 1	1.327340199	0.254401	0.009209	0.447711
Ambra1	Ambra1 Autophagy/beclin 1 regulator 1	1.228958126	0.416754	0.001683	0.177829
App	App Amyloid beta (A4) precursor protein	1.60474612	0.034567	0.154681	0.494304
Atg10	Atg10 Autophagy-related 10 (yeast)	1.425749629	0.023084	0.014835	0.256999
Atg12	Atg12 Autophagy-related 12 (yeast)	1.196807825	0.674936	0.320318	0.123725
Atg16l1	Atg16l1 Autophagy-related 16-like 1 (yeast)	1.483233049	0.204173	0.056958	0.016263
Atg16l2	Atg16l2 Autophagy related 16 like 2 (S. cerevisiae)	1.245718812	0.105069	0.002388	0.256191

Atg3	Atg3 Autophagy-related 3 (yeast)	1.281709031	0.043529	1.018364	0.074163
Atg4a	Atg4a Autophagy-related 4A (yeast)	1.179714413	0.365416	0.038879	0.125473
Atg4b	Atg4b Autophagy-related 4B (yeast)	1.051028754	0.835466	0.031047	0.047305
Atg4c	Atg4c Autophagy-related 4C (yeast)	1.340569337	0.108125	0.003613	0.241178
Atg4d	Atg4d Autophagy-related 4D (yeast)	1.178258062	0.295444	0.002987	0.284877
Atg5	Atg5 Autophagy-related 5 (yeast)	1.464541801	0.021238	0.01512	0.01482
Atg7	Atg7 Autophagy-related 7 (yeast)	1.757102179	0.033926	0.004523	0.01853
Atg9a	Atg9a Autophagy-related 9A (yeast)	1.583941338	0.078667	0.01268	0.09882
Atg9b	Atg9b ATG9 autophagy related 9 homolog B (S. cerevisiae)	6.014516821	0.001354	0.013333	0.040475
Bad	Bad BCL2-associated agonist of cell death	1.574183063	0.07013	0.006672	0.300996
Bak1	Bak1 BCL2-antagonist/killer 1	1.266228163	0.074049	0.063784	0.494212
Bax	Bax Bcl2-associated X protein	2.057840745	0.00171	0.169633	0.21892
Bcl2	Bcl2 B-cell leukemia/lymphoma 2	1.112144673	0.431891	0.002189	0.382453
Bcl2l1	Bcl2l1 Bcl2-like 1	1.915531535	0.025244	0.036572	0.103121
Becn1	Becn1 Beclin 1, autophagy related	1.34217958	0.088816	0.188975	0.186299
Bid	Bid BH3 interacting domain death agonist	1.286287008	0.222125	0.02754	0.414679
Bnip3	Bnip3 BCL2/adenovirus E1B interacting protein 3	1.085133749	0.682742	0.106987	0.113609
Casp3	Casp3 Caspase 3	1.157902293	0.402601	0.019106	0.157401
Casp8	Casp8 Caspase 8	1.307296033	0.020224	0.105532	0.061667
Cdkn1b	Cdkn1b Cyclin-dependent kinase inhibitor 1B	1.295627957	0.00797	0.051723	0.221934
Cdkn2a	Cdkn2a Cyclin-dependent kinase inhibitor 2A	1.375764041	0.043348	0.231044	0.329122
Cln3	Cln3 Ceroid lipofuscinosis, neuronal 3, juvenile (Batten, Spielmeyer-Vogt disease)	1.489875867	0.012428	0.033266	0.167903
Ctsb	Ctsb Cathepsin B	1.273471195	0.081674	0.259226	0.212809
Ctsd	Ctsd Cathepsin D	2.580733772	0.001128	0.174647	0.071874
Ctss	Ctss Cathepsin S	0.941888961	0.85147	0.003004	0.006181
Cxcr4	Cxcr4 Chemokine (C-X-C motif) receptor 4	1.59861585	0.357793	0.007247	0.009969
Dapk1	Dapk1 Death associated protein kinase 1	39.98316719	0.003175	0.002045	0.100198
Dram1	Dram1 DNA-damage regulated autophagy modulator 1	1.23292354	0.311832	0.014585	0.221131
Dram2	Dram2 VDNA-damage regulated autophagy modulator 2	1.042385441	0.863548	0.122662	0.083061
Eif2ak3	Eif2ak3 Eukaryotic translation initiation factor 2 alpha kinase 3	1.506653863	0.002257	0.004903	0.479695
Eif4g1	Eif4g1 Eukaryotic translation initiation factor 4, gamma 1	1.309559236	0.069926	0.086649	0.258593

Esr1	Esr1 Estrogen receptor 1 (alpha)	2.558243166	0.01055	0.00153	0.000873
Fadd	Fadd Fas (TNFRSF6)-associated via death domain	1.766063929	0.0024	0.018152	0.132465
Fas	Fas Fas (TNF receptor superfamily member 6)	1.756495976	0.000857	0.030314	0.455572
Gaa	Gaa Glucosidase, alpha, acid	1.352835291	0.03951	0.023321	0.395567
Gabarap	Gabarap Gamma-aminobutyric acid receptor associated protein	1.577727143	0.003181	4.32585	0.076842
Gabarapl1	Gabarapl1 Gamma-aminobutyric acid (GABA) A receptor-associated protein-like 1	0.99642974	0.978842	0.070326	0.020267
Gabarapl2	Gabarapl2 Gamma-aminobutyric acid (GABA) A receptor-associated protein-like 2	1.615659046	0.000495	0.626835	0.014046
Hdac1	Hdac1 Histone deacetylase 1	1.257022171	0.060447	0.128535	0.411764
Hdac6	Hdac6 Histone deacetylase 6	1.069122585	0.684363	0.021849	0.359177
Hgs	Hgs HGF-regulated tyrosine kinase substrate	1.099562183	0.606127	0.002232	0.153286
Hsp90aa1	Hsp90aa1 Heat shock protein 90, alpha (cytosolic), class A member 1	1.290278748	0.069164	3.212159	0.047711
Hspa8	Hspa8 Heat shock protein 8	1.326943664	0.017792	4.235619	0.282432
Htt	Htt Huntingtin	1.517617176	0.085919	0.016382	0.058418
Igf1	Igf1 Insulin-like growth factor 1	0.726060141	0.023102	0.015319	0.240725
Ins2	Ins2 Insulin II	1.340773171	0.372231	0.005285	0.011126
Irgm1	Irgm1 Immunity-related GTPase family M member 1	1.672913645	0.000162	0.134292	0.234999
Lamp1	Lamp1 Lysosomal-associated membrane protein 1	1.387001135	0.002644	1.450752	0.107813
Map1lc3a	Map1lc3a Microtubule-associated protein 1 light chain 3 alpha	1.42159875	0.023682	0.011569	0.439651
Map1lc3b	Map1lc3b Microtubule-associated protein 1 light chain 3 beta	1.365329598	0.030471	0.682735	0.082414
Mapk14	Mapk14 Mitogen-activated protein kinase 14	1.287663501	0.096587	0.025889	0.111233
Mapk8	Mapk8 Mitogen-activated protein kinase 8	1.066681037	0.645991	0.084303	0.035607
Mtor	Mtor Mechanistic target of rapamycin (serine/threonine kinase)	1.172830442	0.181199	0.007716	0.229621
Nfkb1	Nfkb1 Nuclear factor of kappa light polypeptide gene enhancer in B-cells 1, p105	0.86134866	0.215631	0.030293	0.189956
Npc1	Npc1 Niemann Pick type C1	1.519586063	0.109928	0.056143	0.018579
Pik3c3	Pik3c3 Phosphoinositide-3-kinase, class 3	1.634336738	0.010575	0.030809	0.24255
Pik3cg	Pik3cg Phosphoinositide-3-kinase, catalytic, gamma polypeptide	1.25648993	0.362266	0.028767	0.066656
Pik3r4	Pik3r4 Phosphatidylinositol 3 kinase,	1.312284879	0.047011	0.019348	0.006781

	regulatory subunit, polypeptide 4, p150				
Prkaa1	Prkaa1 Protein kinase, AMP-activated, alpha 1 catalytic subunit	1.273955047	0.024822	0.121013	0.090569
Pten	Pten Phosphatase and tensin homolog	1.605137955	0.00352	0.26807	0.060306
Rab24	Rab24 RAB24, member RAS oncogene family	1.400002233	0.010021	0.075073	0.198763
Rb1	Rb1 Retinoblastoma 1	1.723245533	0.045258	0.022524	0.314655
Rgs19	Rgs19 Regulator of G-protein signaling 19	0.985581106	0.883557	0.054465	0.094282
Rps6kb1	Rps6kb1 Ribosomal protein S6 kinase, polypeptide 1	1.300832513	0.203962	0.230742	0.003843
Snca	Snca Synuclein, alpha	0.979739715	0.794658	0.006582	0.05751
Sqstm1	Sqstm1 Sequestosome 1	0.836892275	0.336786	0.726911	0.002866
Tgfb1	Tgfb1 Transforming growth factor, beta 1	1.402128686	0.009127	0.030374	0.128108
Tgm2	Tgm2 Transglutaminase 2, C polypeptide	1.683038425	0.011647	0.012388	0.034136
Tmem74	Tmem74 Transmembrane protein 74	0.784957053	0.247943	0.017321	0.05757
Tnf	Tnf Tumor necrosis factor	1.204004432	0.797815	0.000472	0.135775
Tnfsf10	Tnfsf10 Tumor necrosis factor (ligand) superfamily, member 10	1.960977355	0.023288	0.008546	0.011834
Trp53	rp53 Transformation related protein 53	1.175304136	0.037463	0.326284	0.052572
Ulk1	Ulk1 Unc-51 like kinase 1 (C. elegans)	1.387537296	0.016975	0.01695	0.07797
Ulk2	Ulk2 Unc-51 like kinase 2 (C. elegans)	1.123750385	0.339582	0.012785	0.478719
Uvrag	Uvrag UV radiation resistance associated gene	1.139726986	0.444456	0.013047	0.394375
Wipi1	Wipi1 WD repeat domain, phosphoinositide interacting 1	1.203542904	0.288162	0.009851	0.495238

Supplementary Table 7. KLF4 manipulation alters expression of a broad spectrum of genes in the autophagy pathway. MEFs were infected with adenovirus expressing KLF4 mRNA or KLF4-targeting shRNA for 72 h. Corresponding empty viruses (Ad-EV, Sh-EV) were used as control. Table shows fold changes and p-values for each gene assessed. ($p<0.05$ by Student T-test, N=3).

**Gene**	**Forward primer 5’-3’**	**Reverse primer 5’-3’**
Epg-2	tcgtcagcagaggatcaaga	gccagcattttgtccaagtt
Atg-2	ctcaaccacatggtgtcgtc	catcggtatggaaagtaacacca
Epg-4	ttcaggagcctgtatgagagc	agcgcagaaatgagagttcc
Unc-51	aaaagggcatcgtacatcgt	attttgggtgcgggagtt
Vps34	aaccctgtcagaaggttgaatc	tgacgagcaagttgagagga
Atg16.1	cagaagttgctttagaagaaaaacg	ttttgtgtccttgtcggtga
Epg-1/atg-13	atccagcaaatggaaccaag	tggagttgattttggagaattg
Pha-4	ctacaccaacggagtataccagaa	ggagaacgtgtgaattggaga
Atg-7	tcttgctatttctgcagtgatgt	gttcctggtcgtgcaacag
Atg-9	ggtcttcacgatgagagtattatcc	tgcatgttgaagcttgacg
Vps15	cgatcgattgagcacgag	tgaagagcaggaagatgtacca
Epg-3	cgagtgtcagagcctggatt	ctttttgttgaggggcattg
Epg-5	gcgccaggattagtagtcaag	ccaattgaggccaatgagtt
Epg-9	gaagtcagttgtgagcagattga	tggcttcttccacttcacatt
Klf-1	cgagctaacaaggcactatagga	Tggcgtttcatatggagtga
Klf-2	tcattttcatacgggacctgtt	gtttgttggacatggttatgatg
Klf-3	catcgccaaacatttttcag	Gcgtcgttcaatcgcttc
P16	gggttttcttggtgaagttcg	ttgcccatcatcatcacct
P21	tccacagcgatatccagaca	ggacatcaccaggattggac

Supplementary Table 8. List of qRT-PCR primers

Gene	Forward primer 5’-3’	Reverse primer 5’-3’
GATA6	tgttgaactgtgcagcttttct	taatgatgcacacacaacctga
BECN1	gcttccaatttgggtggata	aatactgggcaaggcatcat
LC3B	gggaagagccacaagatcag	tcattccccttcagtccttg
ULK1	aactgtgggcagagcctaga	gccatcatgcctagtcacct
ATG7	gctcatgacttcctgttgct	caatgggctgtgactgcaag
ATG9	ttttcctgggtgtgtgcttg	acaaaacacaacatccccact
CTSD	cgtagaagcagcgcatagtc	ctctagccctcttctgtgca
GABARAPL1	ccttctctggacgtttagcc	gatggacctcaggatgtaggg
PIK3C3	cttcttgcttctgtacccgc	gggcgactcagtctatcgg

Supplementary Table 9. List of ChIP-qPCR primers
