# Supplementary material for: A conserved KLF-autophagy pathway modulates nematode lifespan and mammalian age-associated vascular dysfunction
Source: Nat Commun. 2017 Oct 13;8:914. doi: 10.1038/s41467-017-00899-5 (PMC5640649; doi:10.1038/s41467-017-00899-5)
Supplement: Supplementary file 2 — Description of Additional Supplementary Files [file 41467_2017_899_MOESM2_ESM.docx]

**Description of Additional Supplementary Files**

File Name: Supplementary Movie 1

Description: Locomotion behavior of a N2 nematode aged 1 day

File Name: Supplementary Movie 2

Description: Locomotion behavior of a N2 nematode aged 5 days

File Name: Supplementary Movie 3

Description: Locomotion behavior of a N2 nematode aged 9 days

File Name: Supplementary Movie 4

Description: Locomotion behavior of a klf-3 o/e nematode aged 1 day

File Name: Supplementary Movie 5

Description: Locomotion behavior of a klf-3 o/e nematode aged 5 days

File Name: Supplementary Movie 6

Description: Locomotion behavior of a klf-3 o/e nematode aged 9 days
